# Longstanding behavioural stability in West Africa extends to the Middle Pleistocene at Bargny, coastal Senegal

**DOI:** 10.1038/s41559-023-02046-4

**Published:** 2023-05-04

**Authors:** Khady Niang, James Blinkhorn, Mark D. Bateman, Christopher A. Kiahtipes

**Affiliations:** 1 Département d’Histoire, Université Cheikh Anta Diop de Dakar, Dakar, Senegal; 2 Pan-African Evolution Research Group, Max Planck Institute for the Science of Human History, Jena, Germany; 3 Centre for Quaternary Research, Department of Geography, Royal Holloway, University of London, Egham, UK; 4 Department of Geography, University of Sheffield, Sheffield, UK; 5 Institute for the Advanced Study of Culture and the Environment, University of South Florida, Tampa, FL USA

**Keywords:** Archaeology, Palaeoecology

## Abstract

Middle Stone Age (MSA) technologies first appear in the archaeological records of northern, eastern and southern Africa during the Middle Pleistocene epoch. The absence of MSA sites from West Africa limits evaluation of shared behaviours across the continent during the late Middle Pleistocene and the diversity of subsequent regionalized trajectories. Here we present evidence for the late Middle Pleistocene MSA occupation of the West African littoral at Bargny, Senegal, dating to 150 thousand years ago. Palaeoecological evidence suggests that Bargny was a hydrological refugium during the MSA occupation, supporting estuarine conditions during Middle Pleistocene arid phases. The stone tool technology at Bargny presents characteristics widely shared across Africa in the late Middle Pleistocene but which remain uniquely stable in West Africa to the onset of the Holocene. We explore how the persistent habitability of West African environments, including mangroves, contributes to distinctly West African trajectories of behavioural stability.

## Abstract (web summary)

The late Middle Pleistocene site of Bargny, Senegal, documents stone tool trends seen across contemporary sites in Africa but which, in West Africa, remain uniquely stable into the Holocene. Palaeoenvironmental data suggest that persistently stable environments in West Africa through the Late Pleistocene, including estuarine refugia, may have supported consistent behavioural responses.

## Main

The appearance of the Middle Stone Age (MSA) during the Middle Pleistocene (Chibanian; 780–130 thousand years ago (ka)) marks a major cultural change in African prehistory. The earliest occurrence of MSA technologies around 300 ka is broadly contemporaneous with the appearance of *Homo sapiens* in the fossil record^1,2^. Recent studies have highlighted the substantial influence of population structure within and between regions in the mosaic appearance of *Homo sapiens* morphology and subsequent patterns of population diversity^3,4^. The spatial and temporally disjunctive nature of the transition from preceding Acheulean technologies to the appearance of the MSA supports a comparable scenario for cultural evolution, perhaps best evidenced by the late persistence of Acheulean populations at Mieso (Ethiopia)^5,6^ around 60 ka after the appearance of MSA technologies elsewhere in eastern Africa^2^. The transition to MSA technologies is marked by a change in the dominant focus of lithic-reduction practices from production of larger bifacial tools to prepared core reduction and use of retouched flake tool kits, notable increases in transport of raw materials across the landscape and the appearance of additional categories of artefacts within archaeological assemblages, such as ochre^2^. Examining the early appearances of the MSA within the Middle Pleistocene is critical to investigate regional trajectories of behavioural change within their palaeoecological context and inter-regional connectivity in both behaviour and environmental adaptation that complement studies of demographic variability.

**Fig. 1:**
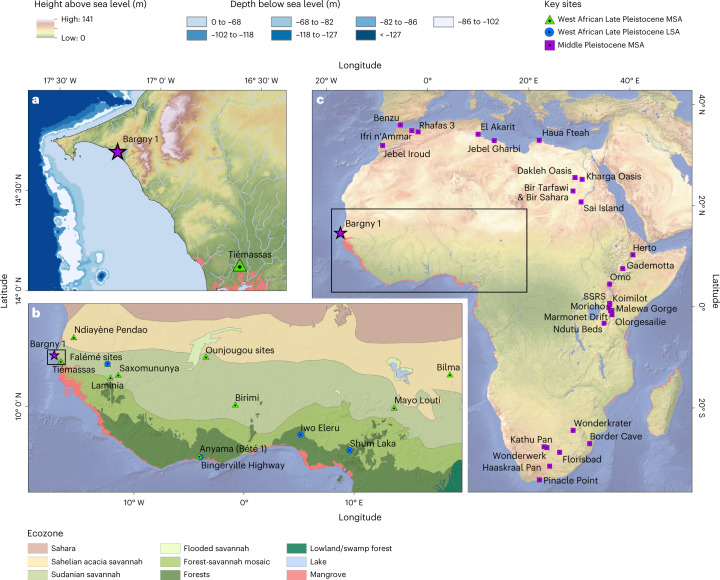
Maps illustrating the location of Bargny 1. **a**, The location of Bargny 1 with respect to the topography and bathymetry of the Senegalese coastline, associated with occupations at Bargny (−86 m to −102 m (white)) and Tiémassas (25 ka: −118 m to −127m; 40–50 ka: −86 m to −68 m; and 62 ka: −86 m to −82 m), Bargny was never more that 26 km from the Pleistocene shoreline (following ref. ^88^). **b**, The distribution of key Late Pleistocene Stone Age sites in West Africa with respect to modern ecozones. **c**, The distribution of Middle Pleistocene MSA sites across Africa. The presence of mangroves is highlighted in pink on all panels (following ref. ^89^). SSRS, Sibilo Road school site. Data in **a** from ALOS (JAXA) and GEBCO 2019 Grid^90^ and in **b** from WWF^91,92^. Map in **c** made with Natural Earth free vector and raster map data (naturalearthdata.com). Figures produced using ESRI ArcMap 10.5.

The earliest occurrences of MSA sites across Africa appear in Marine Isotope Stage (MIS) 9 (337–300 ka), and the initial stages of MIS 8 (300–243 ka) in northern Africa (Jebel Irhoud^7^), eastern Africa (Ologesailie^2^; Gademotta^8,9^) and southern Africa (Kathu Pan^10^). Early-MSA occupations in Africa become more numerous in MIS 7 (243–191 ka) and to a lesser extent in MIS 6 (191–130 ka; Fig. 1); however, our understanding of the MSA in the Middle Pleistocene is heavily biased to the most intensively studied regions of the continent. Across Africa, substantial changes are seen in the MSA from the Late Pleistocene onwards^11–15^, appearing in a mosaic fashion^16^, and reflecting regionalized trajectories of cultural evolution. These include use of alternate lithic-reduction strategies (including a focus on hunting weaponry^17,18^ and heat treatment of lithics^19^), a substantial elaboration of material activity (including burial of the dead^20^, use of organic materials^21^, the production of paint^22^, beads^23,24^ and complex geometric designs^25^) and the occupation of a broader range of habitats^14^, including desert^11,26,27^, high altitudes^28,29^, tropical forests^30,31^ and coastlines^32–34^. Establishing the antiquity of the MSA in poorly understood regions such as West Africa is critical to assess both the earliest appearance of similar novel behaviours, to provide a benchmark against which to evaluate subsequent regionalized patterns of cultural innovation and change and to examine how MSA populations engaged with and adapted to distinct ecological settings.

The chronology of the West African MSA has only recently begun to come into focus^35–38^. The early stages of the MSA in West Africa remain poorly evidenced, with only a terminus post quem date of 254 ka for the Sangoan occupation of Anyama^39^ and terminus ante quem ages from Ounjougou potentially indicative of Middle Pleistocene occupations^40^. In contrast to other regions of the continent, directly dated MSA occupations in West Africa have been restricted to the Late Pleistocene, with the early-MSA assemblage from Ravin Blanc 1 dating to 128–124 ka (ref. ^36^), indicating occupation of the region from the start of MIS 5 (130–71 ka). The majority of MSA sites in the region date between MIS 4 (71–59 ka) and MIS 2 (28–11 ka) (refs. ^35,38,41^), with the youngest MSA occupation across Africa reported from the onset of the Holocene from Saxomununya, about 11 ka (ref. ^37^). West African MSA sites are typically found in modern Sahel or savannah habitats associated with major river basins. The occupation of the site at Tiémassas at the margins of the Saloum delta is a notable exception^38,42^, although direct proxies for palaeoenvironments are typically absent. At Ravin Blanc 1, Douze et al.^36^ question whether the association of crude bifaces with Levallois technologies marks a local post-Acheulean transitional industry, an un-precedented technology that may relate to the Sangoan or simply an early-MSA tradition. Resolving between these alternative patterns of cultural evolution demands the identification of robustly dated Middle Pleistocene MSA assemblages from West Africa. Given the region harbours substantial population diversity, both amongst modern inhabitants^43^ and Late Pleistocene populations^44^, evaluating the early MSA in West Africa may help elucidate relationships between biological structure, behavioural diversity and ecological adaptation.

To explore early-MSA occupations in West Africa, we re-investigated the site of Bargny (Senegal), located in a quarry 1.9 km from the modern shoreline and approximately 30 km east of Dakar (14.7° N, 17.2° W). The site currently lies within the Sudanian vegetation zone (Fig. 1b), a transitional zone largely composed of wooded and tall-grass savannah between the Guinean forests to the south and Sahelian short-grass savannahs to the north^45^. Littoral and coastal vegetation in the region includes Guinean mangroves in estuarine zones and halophytic (salt-tolerant) scrub in dune and coastal plain settings. The modern setting at the site is halophytic scrub situated less than a kilometre from saline marshes and ancient river channels. Today, the region receives an average annual precipitation of 400–500 mm (ref. ^46^). The presence of Stone Age material at the site was initially reported in 1941 by Mauny and Corbeil, who considered it an open-air surface site. Excavations conducted in 1975 reported a shallow stratigraphy of 25 cm (ref. ^47^). We have conducted renewed investigations at the site, enabling us to place a large MSA stone tool assemblage within its chrono-stratigraphic and palaeoecological setting. Here we describe the results of investigation at the site designated Bargny 1 and place them within their wider palaeoanthropological context.

## Results

We investigated the sediment sequence within a quarry pit that had exposed the local bedrock, comprising undulating banded limestones, cleaning the existing section before excavating a 1.75 × 1 m trench in artefact-bearing deposits, to recover lithic artefacts and sediment samples for further analysis (Methods).

### Stratigraphy

**Fig. 2:**
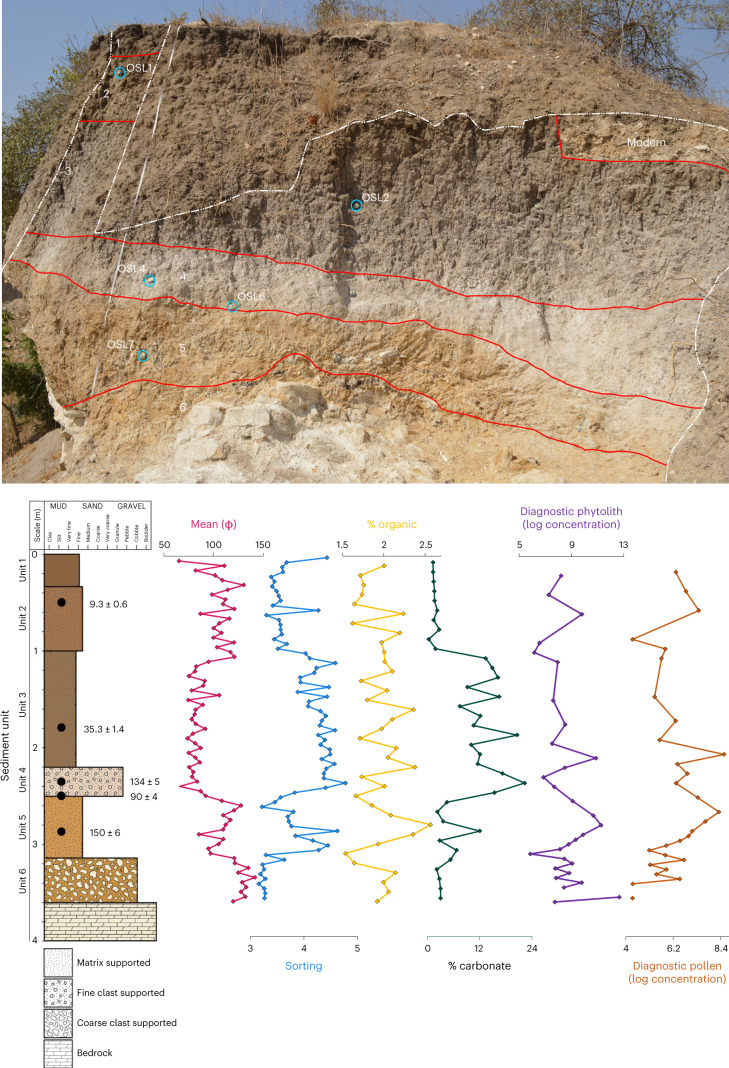
Photo of sediment section at Bargny 1 and the results of geochronological, sedimentological and palaeoecological analyses. Top, photo of excavated and sampled section at Bargny 1 (outlined in white), illustrating the division of major sediment units (red) and the locations of dating samples (blue). Bottom left, log diagram of sediment units, illustrating relative position of geochronological samples (black dots) with results of sedimentological and palaeoecological studies including mean fine-sediment particle size (pink; nm), sorting (blue), total organic matter (%; yellow), carbonates (%; green), diagnostic phytoliths (log-transformed concentration, purple) and diagnostic pollen (log-transformed concentration, orange).

Six major sediment units were identified in the field and further resolved through laboratory analyses (Fig. 2). The top unit (Unit 1) is the dark-brownish-grey silty sand modern topsoil and appears broadly level across the landscape, underlain by Unit 2, a mid-greyish-brown silty sand sub-soil, and combined, they span the upmost 1 m of the deposit. A sharp contact is observed with the underlying deposit (Unit 3) that is comprised of a mid-greyish silty sand with occasional limestone grits (~5 mm in diameter). A further sharp contact is observed at about 2.2 m below the surface with Unit 4, a fine gravel that is typically 35 cm thick and composed of limestone grits (5–10 mm in diameter) supporting a pale-brownish-grey silty sand. The presence of limestone grits is best explained by the erosion of local limestone sources in the immediate vicinity of the site that comprise bedrock. Both a change in sediment texture and colour mark a sharp contact with the lowermost phase of sedimentation at about 2.5 m below the surface, with the presence of Unit 5, a pale-orange silty sand with occasional limestone grits and lithic artefacts and Unit 6, a clast-supported pale-orange silty sand with rare limestone gravels (up to 20 mm in diameter) and clasts comprising sub-angular fragments of chert or the underlying limestone bedrock. The prominent orange colour of fine sediments in units 5 and 6 may be attributed to transportation of eroded products from iron crust deposits, which are found to cap limestone deposits elsewhere along the coastline but are not evident in the immediate vicinity of the site.

### Geochronology

We recovered five samples for dating from Units 1 (0.45 m), Unit 3 (1.9 m), Unit 4 (2.6 m), the interface of Units 4 and 5 (2.8 m) and Unit 5 (3.1 m). We dated coarse quartz grains with optically stimulated luminescence (OSL) at the single-aliquot level, with all samples possessing good luminescence characteristics, OSL signals dominated by a fast component, low levels of palaeodose (D_e_) overdispersion and high D_e_ reproducibility (Methods and Supplementary Information 1). The results of OSL dating are presented in Table 1.

**Table 1:** Results of OSL dating at Bargny 1

Lab code	Depth (m)	Unit	*N*	D_e_ (Gy)	OD^a^ (%)	Dose rate (µGy a^−^^1^)	Age (ka)	MIS age
Shfd19079	0.45	1	24 (22)	9.7 ± 0.5	25 (21)	1,045 ± 40	9.3 ± 0.6	1
Shfd19080	1.9	3	23 (20)	40.1 ± 0.3	7 (2)	1,135 ± 45	35.3 ± 1.4	3
Shfd19081	2.6	4	21 (21)	108 ± 1	5 (5)	813 ± 32	134 ± 5^b^	5
Shfd19082	2.8	4/5	23 (22)	84 ± 1	14 (8)	896 ± 37^a^	93 ± 4^b^	5
Shfd19083	3.1	5	21 (17)	126 ± 2	11 (6)	840 ± 33	150 ± 6	6

^a^Overdispersion (OD) on the Central Age Model with values in parenthesis calculated after outlier removed.

^b^Unit 4 is carbonate rich (limestone). Dose rate and ages for this unit assume erosion of local limestone and deposition of it as clasts at the same time as quartz deposition and burial.

Palaeodose (D_e_) values from individual aliquots were accepted only if they exhibited an OSL signal measurable above background, good growth with dose, recycling values within ± 10 % of unity and the error on the test dose used within the SAR protocol was less than 20%. The number of aliquots accepted from the 24 measured (*N*) is shown.

OSL results indicate that sediment aggradation associated with Unit 5 that preserved evidence of MSA occupation began during the latter half of MIS 6. Sediments were primarily sourced from the erosion of ferricrete formations. While obtaining an indicative MIS 5 age for the overlying Unit 4, the presence of an age reversal between the two samples means that we must be cautious with ages from this unit. Sedimentologically and geochemically, Unit 4 is internally variable and notably different to other units (Methods and Supplementary Information 1). Field observations suggest the relatively high carbonate content is not of post-depositional pedogenic or groundwater origin. Instead, field observations determine that local limestone was eroded and deposited as clasts within Unit 4 at the same time as the silty sand, meaning the age reversal cannot be explained by uncorrected changing carbonate contents through time impacting on the OSL dose rate. The modern land surface appears to have been established in the early Holocene.

### Palaeoecology

Present-day Bargny vegetation consists of grassland with few shrubs dominated by species such as *Acacia seyal* and *Calotropis procera*. For past vegetation reconstruction, we evaluated sediment samples for microbotanical remains (phytoliths and pollen; Supplementary Information 2). A targeted subset of 24 samples yielded phytolith concentrations suitable for palaeoecological analysis. The abundance of plant microfossils closely follows the major sedimentary units. Units 1, 2 and 3 have fewer microfossils and poor preservation while Units 4, 5 and 6 show phases of enhanced microfossil deposition and preservation consistent with the greater organic content and sediment sorting observed in the sedimentology (Fig. 2). Pollen concentration is highly variable, and preservation is low, which limits the utility of quantitative analysis, but the presence of locally distributed wetland indicators (*Typha*, *Avicennia*) (Supplementary Fig. 2.1) and regional pollen arriving from the jet stream (Pinaceae) contextualizes the phytolith results in important ways. We evaluated the representativeness of the Bargny phytolith results using comparisons with modern surface samples^48,49^. Both PCA (Supplementary Fig. 2.6) and minimum square-chord distances (Supplementary Fig. 2.7) show that sedimentary phytolith signatures fall within the envelope of phytolith signals from modern West African vegetation zones. We also used indices of grass–water stress (Fst; ref. ^48^) and grass composition (Iph; refs. ^48,50^) to characterize past vegetation cover at Bargny (Supplementary Fig. 2.8).

Phytoliths from Units 1, 2 and 3 show a stronger signal of water stress, a higher ratio of short- to long-grass phytoliths and are most similar to modern samples from the Sahelian and Saharan vegetation zones (Supplementary Figs. 2.14 and 2.15). Pollen recovered from this section shows a strong representation of halophytic scrub (Amaranthaceae) but also includes pollen from cattail (*Typha*) and sedge (Cyperaceae), which are common in the brackish wetlands located within 1 km of the site. This agrees with the regional pattern of arid conditions spanning the end of MIS 3 to the onset of the Holocene. Water stress and short-grass indices are tightly constrained around low values in units 4 and 5 (Supplementary Fig. 2.18). Phytoliths from these samples are most similar to surface samples from wetter varieties of Sahelian and Sudanian vegetation with annual precipitation above 500 mm per year. Low grass-stress values persist in Unit 6, but the short grass index values are extremely variable. Low water stress and short grass indices are out of sync with expected conditions during MIS 6, which is associated with enhanced jet stream circulation, low precipitation and a southward shift of the Sahelian–Saharan boundary^51,52^. Pollen in these units includes types introduced by jet stream activity (Pinaceae) and typical brackish mangrove taxa (*Avicennia*, *Typha*). Supported by the sedimentological results, we interpret the phytolith and pollen signal to indicate that estuarine conditions persisted at Bargny during the latter half of MIS 6, probably supported by local hydrogeological conditions and spring activity, which are common in the modern landscape around the site.

### Stone tool technology

**Fig. 3:**
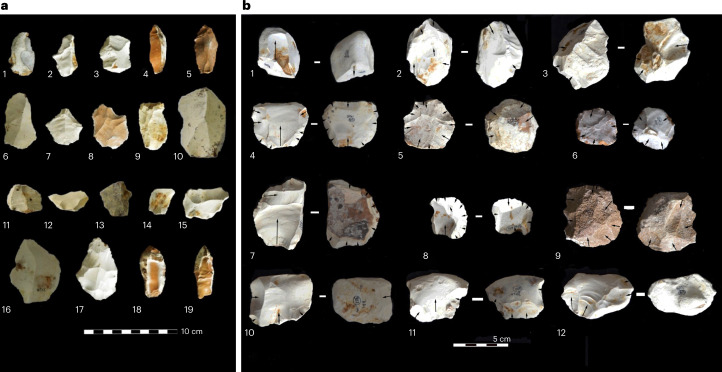
Photos of Middle Pleistocene MSA artefacts from Bargny 1. **a**, Flakes: cortical flakes (1, 2, 3) cortical blade (4), blade (5), levallois flakes (6, 7, 8, 9), levallois point (10), pseudo-Levallois point (11, 12, 13), flakes (14, 15, 16), retouched flakes (17, 18, 19). **b**, Cores (with diacritic markings of flake removals): single-platform (1), multiple-platform core (2, 3), Levallois preferential core (4), Levallois recurrent centripetal (5, 6), Levallois core fragment (7), discoidal core (9), core-on flake (8, 10, 11), single-platform core-on plaquette (12).

A stone tool assemblage (*n* = 772) was recovered from Unit 5, which represents all stages of lithic reduction, suggesting on-site activity (Fig. 3 and Table 2). Artefacts were uniformly distributed in the excavated deposits and visible in section across the landscape where Unit 5 deposits were exposed, with no evidence to suggest occupations in overlying deposits at this locality. High-quality chert yielded by local limestone has been used to produce the majority of artefacts (~98%), directly present at the site in the underlying bedrock and clasts of varying morphology within Unit 6, with rare artefacts made from sandstone and quartz suggesting transport to the site from more distant locations. The artefact assemblage indicates the dominance of Levallois reduction strategies across cores and blanks and supported by the presence of related core-management and trimming elements associated with the repreparation of debitage surfaces. Unidirectional Levallois schemes, including point and blade production, are present, but centripetal Levallois flaking strategies predominate, evident across preferential and recurrent reduction schemes. Unifacial discoidal flaking is rarer but evident from the presence of four cores and six pseudo-Levallois points. High intensity of reduction activity is evident in the conspicuous presence of core-on flakes (*n* = 15) and core-on-flake fragments (*n* = 6), with unipolar, orthogonal and centripetal scars present. Informal core reduction is evident from the presence of multi-platform cores and fragments (*n* = 27) and single-platform cores and fragments (*n* = 6). Retouched artefacts are extremely scarce, comprising seven scrapers. This stone tool assemblage is characteristic of both regional and continental expressions of MSA lithic technology.

**Table 2:** Stone tool artefacts recovered by excavation at Barny 1, split into key technological groups

Group	Category	Number	Percentage
Complete cores	Single-platform core	2	0.26
	Multiple-platform core	9	1.16
	Levallois core	16	2.07
	Discoidal core	2	0.26
	Core-on flake	9	1.16
	Subtotal	38	4.92
Complete debitage	Core management	16	2.07
	Core-trimming element	11	1.42
	Flakes	259	33.55
	Blades	23	2.98
	Levallois products	48	6.21
	Pseudo-Levallois point	6	0.78
	Retouched flakes	7	0.90
	Subtotal	371	48.05
Broken pieces	Single-platform core fragment	2	0.26
	Multiple-platform core fragment	9	1.6
	Levallois core fragment	6	1.6
	Discoid core fragment	2	0.12
	Core-on-flake fragment	6	0.77
	Flake fragment	222	28.75
	Hammer	1	0.12
	Debris, indeterminate, parasite flake	115	15.02
	Shatter (140–187 g)		
	Subtotal	363	47.02
Total		772	100

## Discussion

**Fig. 4:**
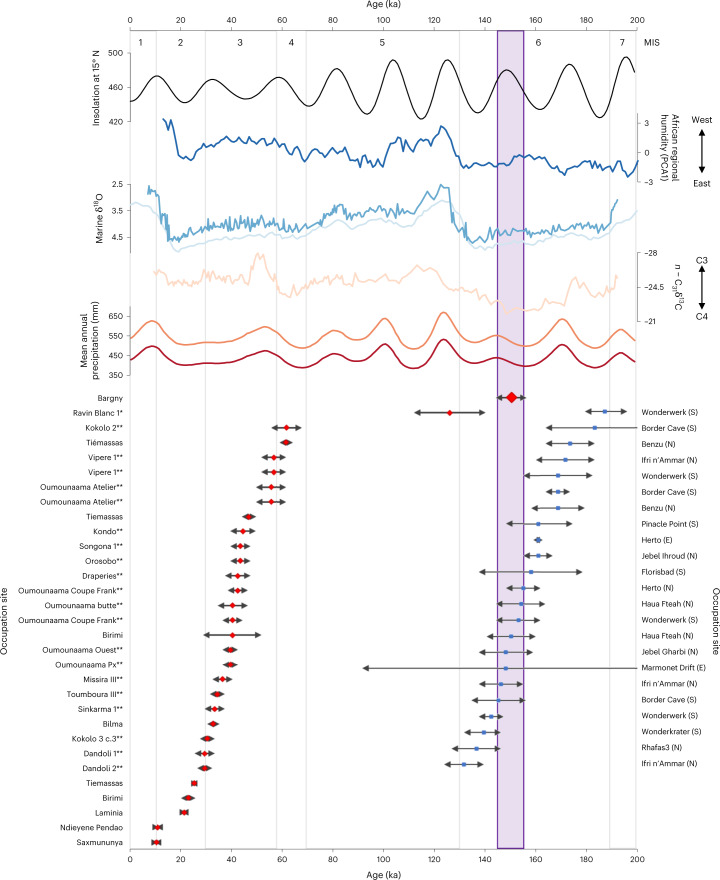
Palaeoenvironmental context of MSA occupations of West Africa. Illustrating mean summer insolation at 15° N (black, following ref. ^93^), inter-regional African humidity (dark blue, PCA1^71^); marine δ^18^O (mid blue, Atlantic stack^94^; light blue, GeoB5928-3^95^), *n*-alkane leaf wax isotopes (pink, C_31_ δ^13^C, GeoB5928-3^95^), modelled mean annual precipitation (orange, within 0.5° cell, red, delta downscaled to 0.925 km on-site location, following refs. ^69,96,97^) and synthesis of dated MSA occupations from West Africa (red diamonds; * Falémé sites; **Ounjougou sites) and other MIS 6 MSA sites across Africa (blue squares; location in N, E, or S Africa shown in parentheses). The occupation at Bargny (highlighted in purple) coincides with a peak in insolation, C4 plants and more arid environments than Late Pleistocene MSA sites in West Africa and precedes a regional shift in humidity from East to West Africa.

Our study at Bargny documents evidence for an MSA inhabitation at 150 ± 6 ka in close proximity to estuarine and semi-arid habitats within about 22 km of the Senegalese coastline and extends the chronology of the West African MSA into the Middle Pleistocene for the first time. We compared the typo-technological composition of Bargny 1 to 23 other MSA assemblages dating to MIS 6, including assemblages from northern Africa (Benzu^53^, Haua Fteah^54,55^, Ifri n’Ammar^56^ and Rhafas^57,58^), eastern Africa (Herto^59^, Marmonet Drift^60^) and southern Africa (Border Cave^61^, Florisbad^62^, Pinacle Point^63,64^, Wonderkrater^65^)(Fig. 4 and Supplementary Table 2.1). Levallois-flake technologies are the most consistent diagnostic feature of other MIS 6 MSA assemblages, occurring in 74% of assemblages, followed by 70% of assemblages containing blade production and 65% containing centripetal-reduction methods, fitting with the dominant reduction approaches at Bargny 1. Retouched points are the most common tool found in other MIS 6 MSA assemblages (78%), which are absent from Bargny (although Levallois-point production is present), with scrapers appearing in 52% of assemblages and other tools, including heavy tools, appearing more sparsely. The assemblage from Bargny 1 is typical of the patterns of typo-technological diversity in early-MSA assemblages across Africa in MIS 6, focusing on Levallois and other radially focused reduction methods and in the absence of elaborate retouched tool kits.

Placed in the wider context of dated MSA stone tool assemblages from West Africa (*n* = 31; Fig. 4 and Supplementary Table 3.2), including Ravin Blanc 1^36^ and the Falémé valley^35,37^, lower Senegal valley^66^, Gambia valley^37^, Ounjougou^35^, Birimi and Bilma^67^ and Tiémassas^38,42^, our results from Bargny extend the time frame in which a stable and enduring suite of lithic-reduction practices persisted in the region, spanning the late Middle Pleistocene to the onset of the Holocene (Fig. 4). A shared focus on Levallois, blade and discoidal reduction and retouched tool kits apparent at Bargny 1 and Ravin Blanc 1, appearing at the very start of MIS 5, characterizes the early MSA in the region. Given these similarities, the scarce presence of bifaces at Ravin Blanc 1 appears unlikely to indicate a discrete transitional industry. Among the 24 dated sites that document reduction technology, centripetal (discoidal/radial; 67%) and Levallois (54%) methods appear as enduring features of West African MSA assemblages, found across a range of sites and remaining present in the region’s youngest MSA assemblage. While blade production is present both at Bargny 1 and Ravin Blanc 1, it appears at low frequency across MSA assemblages in the region (33%). Bipolar reduction similarly appears at low frequencies (29%) but is apparent only in assemblages dating to MIS 4 and 3. Heavy tools appear sparsely in the West African MSA record, occurring in ~23% of assemblages. Retouched tools are reported from 17 of the 31 dated West African MSA assemblages, half of which include a single tool type. Retouched points (65%) and scrapers (53%) are the most common tool types, with other types appearing more sparsely and without clear spatial or temporal patterning. Patterns of core reduction that appear in the early-MSA assemblage at Bargny 1 form a consistent part of later-MSA technology in West Africa, but in contrast to other regions of the continent, there appears to be limited elaboration of stone-reduction methods or retouched tool kits during the Late Pleistocene. Indeed, remarkably close comparability can be observed in the oldest (Bargny 1) and youngest (Saxomununya) MSA stone tool assemblages in West Africa.

Substantive variability can be observed in regional and continental climatic and palaeoecological records spanning the past 200 ka (Fig. 4), and on-site proxy records from Bargny 1 provide an important means to constrain how this variability is manifest at a local level. The development of estuarine conditions from at least 150 ka despite the substantial flux in the limits of arid Saharan and Sahelian vegetation evident in regional marine records^51,52^ may result from coastal hydrogeological processes. Low MIS 6 sea levels exposed a vast coastal plain with low topographic relief where spring-fed streams could support complexes of estuarine systems. The establishment of modern landscape processes in the Late Pleistocene is evident in the turnover in the depositional environment and the appearance of vegetation communities comparable to drier varieties of Sudanian woodlands. Outside of changes in the local depositional environment, the evidence from Bargny 1 is consistent with climate-model outputs (Fig. 4), suggesting local constraints on the scope of climate change. A recent study of human refugia in Africa has highlighted the potential suitability of habitats in Senegal, Gambia and western Mali (referred to hereafter as the Senegambian refugia; Supplementary Information 3) to enable persistent occupation throughout the Late Pleistocene based on precipitation thresholds of 248–1403 mm, based on ethnographic analogies^68^ and corroborated by recent examination of the eastern African MSA record^69^. The distribution of this proposed refugia is consistent with the distribution and chronology of previously known MSA occupations in western Africa. Extending this analysis to cover the time frame from the oldest MSA occupation at Bargny to the youngest at Saxomununya corroborates the persistent presence of precipitation within the 248–1,403 mm threshold for this Senegambian refugia from mid-MIS 6 (160 ka) to the Holocene that is consistent with records from Bargny 1 and across the region (Supplementary Information 3 and Supplementary Fig. 3.1). This refugia appears to be dominated by tropical xerophytic shrubland habitats with limited connectivity to other, more ecologically diverse refugia (such as in northern, eastern or southern Africa^68^. The lack of longstanding connectivity across the region is consistent with genetic studies of contemporary populations in western Africa^70^, which emphasizes extended phases of isolation from other populations and notable archaic introgression. Similarly, study of the only Pleistocene fossil from the region^44^ highlights the potential for complex population structuration in the region. The enduring habitability of the Senegambian refugia and limited inter-regional demographic connectivity could plausibly support the longevity of cultural continuity observed in this region between 150 ka and 10 ka.

The occupation at Bargny around 150 ka highlights that inhabitation of the Senegambian refugia precedes both wider expansions into novel habitats in MIS 5 and particularly across the adjacent arid landscapes of the Sahara^11,12^ and a major change in inter-regional climatic patterns across Africa, with a notable shift from East to West African humidity^71^. This sets an important baseline both for understanding the longevity of occupation of West Africa and the range of climatic settings in which the region has hosted MSA populations, and from which to explore demographic expansions. Evidence for connections between landscapes immediately to the north or south remain limited, however. An individual example of a tanged point is found at Tiémassas, where occupations span MIS 4–2^38,42^, which have traditionally been an important *fossile directeur* for MIS 5 (‘Aterian’) expansions across the Sahara. Understanding of MSA occupations of forested regions to the south remain poorly known in terms of technology and chronology, limiting the scope of comparisons. The archaeological record supports genetic and fossil records that suggesting longstanding population continuities and limited inter-regional contacts. The absence of demographic pressures in the region could have limited the scope for adaptive innovation^72–74^, consistent with the broad technological continuity in the region from the 150 ka occupation of Bargny to the 10 ka occupation of Saxomununya^37^.

The importance of the coastline to Pleistocene populations in tropical Africa has seen highly limited investigation to date. Substantial research has been conducted into patterns of coastal and marine exploitation elsewhere that demonstrate their early importance and integration into wider subsistence patterns. Such studies benefit from extensive research histories on near-coastal cave sites^75^, whereas in tropical Africa, inland sites have been the main focus, even in regions such as eastern Africa that share comparable scales of exploration by Stone Age archaeologists^69^. Panga ya Saidi, on the Kenyan coast, provides evidence of long-term engagement with coastal resources, although distinct from the more-intensive records from northern and southern Africa^34^. In West Africa, the occupation of Bargny supplements evidence from Tiémassas^38,42^, suggesting repeated inhabitation close to the Senegalese coastline spanning MIS 6–2, yet these records do not yet illustrate clear engagement with coastal resources. Relict populations of *Avicennia* still occupy brackish mangroves in infrequently inundated backwaters fringing the West African coastline as far as 19° N (ref. ^76^). Mangroves have high habitat heterogeneity and accessible subsistence resources, making them potential hotspots for Pleistocene forager populations. Adaptation to exploit these resources has the potential to open new axes for population expansions in West Africa^77^. Accelerating research interest in the West African Stone Age provides the means to test such proposals. As new data emerge, we must consider that from at least the end of the Middle Pleistocene, West Africa may have been a source of behavioural and biological diversity, enabling regionally distinct patterns of human evolution through the Late Pleistocene.

## Methods

### Excavation

The excavation site is located within a quarry, where the sediment sequence had not been removed due to its proximity to a site where mystic rituals and practices of a Lebou community were undertaken. Quarry activities had partially exposed the archaeological horizon, enabling us to identify and target the sediment unit of interest. Following cleaning of any sediment disturbed by quarrying activity, we excavated a 1.75 × 1 m area of intact sediment deposit, differentiating between discrete sediment units subdivided into 5 cm arbitrary horizons to control artefact provenance and recovery. All excavated sediments were wet sieved through a 5 mm mesh to maximize artefact recovery. Cleaning of the entire sediment sequence in a sampling column was undertaken before recovering additional sediment samples for sedimentological, geochronological and palaeoecological analyses, with bulk sediment samples recovered at 5 cm intervals.

### Sedimentology

For laser particle-size analysis, sieved sediment samples (~1 g, <2 mm) were bathed for 24 hours in sodium hexametaphosphate (4.4%) solution and agitated in an ultrasonic bath, with samples rinsed in purified water before analysis in a Malvern Mastersizer 3000. Characterization of the grain-size results were conducted using Gradistat. For loss on ignition studies, sediment samples (~10 g) were weighed (to three decimal places, that is, 0.001 g) and heated in a muffle furnace to 105 °C, 550 °C and 950 °C (allowing the sediments to cool to 105 °C for weighing between steps) to calculate the proportions of water, total organic matter, carbonates and mineral residue.

### Geochronology

OSL dose rates were based on concentrations of potassium (K), thorium (Th), uranium (U) and Rubidium (Rb) as determined by inductively coupled plasma mass spectrometry and converted to annual dose rates following Guerin and colleagues^78^ (Table 1). Attenuation of dose by moisture used present-day values with a ± 3% error to incorporate fluctuations through time (Supplementary Table 1.1), and cosmic dose rates were calculated from ref. ^79^. Contributions of gamma dose from adjacent units was modelled using data from ref. ^80^. The impacts on dose rate to possible post-depositional changes to carbonate content (ref. ^81^) were considered (Supplementary Table 1.2). As the carbonate was in the form of limestone clasts deposited at the same time as the dated quartz, we took the view that no correction was necessary (Supplementary Information 1). Future in situ gamma-dose measurements might resolve whether the age reversal is in part due to dose rate over estimation from the unsampled clastic material.

OSL samples were prepared following ref. ^82^. Luminescence measurements of 8 mm in diameter aliquots used a Risø DA-20 luminescence reader. Sample palaeodoses (De) were measured using the single-aliquot regenerative protocol^83^ with an experimentally derived preheat of 260 °C for 10 seconds. Twenty-four replicate aliquots per samples were measured. Sample D_e_ replicates were normally distributed with low overdispersion (Table 1), showing no indication of either partial bleaching or post-depositional disturbance^84^. D_e_ values for age calculation used the Central Age Model^85^.

### Palaeoecology

A subset of sediment samples were selected for processing, focusing on the MSA occupation horizon, and to provide a more general characterization of overlying, culturally sterile deposits. Sediment samples were ground, passed through a 250-micron sieve and placed in a shaker overnight with Calgon solution (sodium hexametaphosphate) before having sands and clays separated by gravity settling and centrifugation–decant cycles at 2,500 r.p.m. for 2 min. At this point, samples were spiked with *Lycopodium* spores and treated with 10% HCl in a 40 °C bath for 10 min. After centrifugation–decant cycles until pH neutral, the samples were separated by density using a solution of zinc bromide and 5% HCl with a specific gravity of 2.3 g ml^−1^. The resulting residue was extracted in ethanol and transferred to glycerol for analysis. Owing to the well-oxidized state of the samples, the use of a strong oxidizing agent was omitted to preserve organic microfossils and enable the use of *Lycopodium* to track laboratory errors and calculate microfossil concentrations. Phytolith and pollen microfossils were identified using a binocular light microscope at 400×–1,000×. Phytolith nomenclature and categories follow the International Code for Phytolith Nomenclature^86^, but we tried specifically to create sample categories consistent with Bremond and colleagues’^48,49^ assessment of phytoliths from surface samples across West Africa.

### Stone tools

Macroscopic evaluation of all lithic artefacts established patterns of raw material use after which artefacts were separated into basic technological categories (cores, flakes, retouched pieces, debris) and identification of fragmented pieces. All specimens were weighed and basic metric attributes recorded (maximum and axial length, width and thickness). Alternate technological approaches (for example, Levallois, discoidal) and blank types (for example, blades, points) were identified through evaluation of flake-scar characteristics, number and formulation of exploited surfaces, nature of flaking platforms and the direction of exploitation from the flaking platform. Comparative analyses of stone tool assemblages from MIS 6 elsewhere in Africa and throughout the West African MSA identified the presence/absence of 16 stone tool forms, including backing, bipolar technology, blade technology, borers, burins, centripetal technology, large cutting tools, denticulates, Levallois blade technology, Levallois-flake technology, Levallois point technology, notches, platform core technology, point technology, retouched bifacial pieces and scrapers, following refs. ^14,69,87^.

### Reporting summary

Further information on research design is available in the Nature Portfolio Reporting Summary linked to this article.

## Supplementary information

Supplementary Information Supplementary Information 1: luminescence dating (including Supplementary Tables 1.1–1.2 and Figs. 1.1–1.3). Supplementary Information 2: plant microfossils (including Supplementary Tables 2.1–2.3 and Figs. 2.1–2.18). Supplementary Information 3: archaeological comparisons and refugia analysis (including Supplementary Tables 3.1–3.2 and Fig. 3.1).

Reporting Summary

## Data Availability

The authors confirm that the data supporting the findings of this study are available within the article, its supplementary information and via Figshare: 10.6084/m9.figshare.22293565. Pollen and phytolith results will also be made available through the African Pollen Database and Neotoma Paleoecology Database.

## Code availability

Raw data and code supporting the findings of palaeoenvironmental reconstruction are publicly available at 10.6084/m9.figshare.22293565.

## References

[1] Hublin J (2017). New fossils from Jebel Irhoud, Morocco and the pan-African origin of Homo sapiens. Nature.

[2] Brooks AS (2018). Long-distance stone transport and pigment use in the earliest Middle Stone Age. Science.

[3] Scerri EML (2018). Did our species evolve in subdivided populations across Africa, and why does it matter?. Trends Ecol. Evol..

[4] Scerri EML, Chikhi L, Thomas MG (2019). Beyond multiregional and simple out-of-Africa models of human evolution. Nat. Ecol. Evol..

[5] De la Torre I, Mora R, Arroyo A, Benito-calvo A (2014). Acheulean technological behaviour in the Middle Pleistocene landscape of Mieso (East-Central Ethiopia). J. Hum. Evol..

[6] Benito-Calvo A, Barfod DN, Mchenry LJ, De I (2014). The geology and chronology of the Acheulean deposits in the Mieso area (East-Central Ethiopia). J. Hum. Evol..

[7] Richter D (2017). The age of the hominin fossils from Jebel Irhoud, Morocco, and the origins of the Middle Stone Age. Nature.

[8] Morgan LE, Renne PR (2008). Diachronous dawn of Africa’s Middle Stone Age: new ^40^Ar/^39^Ar ages from the Ethiopian Rift. Geology.

[9] Douze K, Delagnes A (2016). The pattern of emergence of a Middle Stone Age tradition at Gademotta and Kulkuletti (Ethiopia) through convergent tool and point technologies. J. Hum. Evol..

[10] Porat N (2010). New radiometric ages for the Fauresmith industry from Kathu Pan, southern Africa: implications for the earlier to Middle Stone Age transition. J. Archaeol. Sci..

[11] Scerri EML (2017). The North African Middle Stone Age and its place in recent human evolution. Evol. Anthropol..

[12] Scerri EML, Spinapolice EE (2019). Lithics of the North African Middle Stone Age: assumptions, evidence and future directions. J. Anthropol. Sci..

[13] Tryon CA, Faith JT (2013). Variability in the Middle Stone Age of Eastern Africa. Curr. Anthropol..

[14] Blinkhorn J, Grove M (2018). The structure of the Middle Stone Age of eastern Africa. Quat. Sci. Rev..

[15] Wadley L (2015). Those marvellous millennia: the Middle Stone Age of Southern Africa. Azania: Archaeol. Res. Afr..

[16] Mcbrearty S, Brooks AS (2000). The revolution that wasn’t: a new interpretation of the origin of modern human behavior. J. Hum. Evol..

[17] Lombard M (2021). Variation in hunting weaponry for more than 300,000 years: a tip cross-sectional area study of Middle Stone Age points from southern Africa. Quat. Sci. Rev..

[18] Backwell L (2018). The antiquity of bow-and-arrow technology: evidence from Middle Stone Age layers at Sibudu Cave. Antiquity.

[19] Schmidt P (2015). A previously undescribed organic residue sheds light on heat treatment in the Middle Stone Age. J. Hum. Evol..

[20] Martinón-Torres M (2021). Earliest known human burial in Africa. Nature.

[21] Bouzouggar A (2018). 90,000 year-old specialised bone technology in the Aterian Middle Stone Age of North Africa. PLoS ONE.

[22] Henshilwood CS (2011). A 100,000-year-old ochre-processing workshop at Blombos Cave, South Africa. Science.

[23] Bouzouggar A (2007). 82,000-year-old shell beads from North Africa and implications for the origins of modern human behavior. Proc. Natl Acad. Sci. USA.

[24] Vanhaeren M, Wadley L, d’Errico F (2019). Variability in Middle Stone Age symbolic traditions: the marine shell beads from Sibudu Cave, South Africa. J. Archaeol. Sci. Rep..

[25] Henshilwood CS (2018). An abstract drawing from the 73,000-year-old levels at Blombos Cave, South Africa. Nature.

[26] Burrough SL, Thomas DSG, Barham LS (2019). Implications of a new chronology for the interpretation of the Middle and Later Stone Age of the upper Zambezi Valley. J. Archaeol. Sci. Rep..

[27] Mccall, G. S., Marks, T. P., Thomas, J. T., Iii, S. W. H. & Taylor-Perryman, R. Erb tanks: a Middle and later Stone Age rockshelter in the Central Namib Desert, Western Namibia. *PaleoAnthropology*10.4207/PA.2011.ART67 (2011).

[28] Ossendorf G (2019). Middle Stone Age foragers resided in high elevations of the glaciated Bale Mountains, Ethiopia. Science.

[29] Pazan, K. R., Dewar, G. & Stewart, B. A. The MIS 5a (~80 ka) Middle Stone Age lithic assemblages from Melikane Rockshelter, Lesotho: highland adaptation and social fragmentation. *Quat. Int.*10.1016/j.quaint.2020.11.046 (2020).

[30] Shipton C (2018). 78,000-year-old record of Middle and later stone age innovation in an East African tropical forest. Nat. Commun..

[31] Roberts P (2020). Late Pleistocene to Holocene human palaeoecology in the tropical environments of coastal eastern Africa. Palaeogeogr. Palaeoclimatol. Palaeoecol..

[32] Campmas E (2016). Initial insights into Aterian hunter–gatherer settlements on coastal landscapes: the example of Unit 8 of El Mnasra Cave (Témara, Morocco). Quat. Int..

[33] Will M, Parkington JE, Kandel AW, Conard NJ (2013). Coastal adaptations and the Middle Stone Age lithic assemblages from Hoedjiespunt 1 in the Western Cape, South Africa. J. Hum. Evol..

[34] Faulkner P (2021). 67,000 years of coastal engagement at Panga ya Saidi, eastern Africa. PLoS ONE.

[35] Chevrier B (2018). Between continuity and discontinuity: an overview of the West African Paleolithic over the last 200,000 years. Quat. Int..

[36] Douze K (2021). A West African Middle Stone Age site dated to the beginning of MIS 5: archaeology, chronology, and paleoenvironment of the Ravin Blanc I (eastern Senegal). J. Hum. Evol..

[37] Scerri EML (2021). Continuity of the Middle Stone Age into the Holocene. Sci. Rep..

[38] Niang K (2020). The Middle Stone Age occupations of Tiémassas, coastal West Africa, between 62 and 25 thousand years ago. J. Archaeol. Sci. Rep..

[39] Liubin, V. P. & Guédé, F. Y. *Paleolit Respubliki Kot d’Ivuar (Zapadnaya Afrika)* (Rossiiskaya Akademiya Nauk, 2000).

[40] Soriano, S., Rasse, M., Tribolo, C. & Huysecom, É. in *West African**Archaeology: New Developments, New Perspectives* (ed. Allsworth-Jones, P.) (BAR Publishing, 2010).

[41] Scerri EML, Blinkhorn J, Niang K, Bateman MD, Groucutt HS (2017). Persistence of Middle Stone Age technology to the Pleistocene / Holocene transition supports a complex hominin evolutionary scenario in West Africa. J. Archaeol. Sci. Rep..

[42] Niang K, Blinkhorn J, Ndiaye M (2018). The oldest Stone Age occupation of coastal West Africa and its implications for modern human dispersals: new insight from Tiémassas. Quat. Sci. Rev..

[43] Skoglund P (2017). Reconstructing prehistoric African population structure. Cell.

[44] Harvati K (2011). The later Stone Age calvaria from Iwo Eleru, Nigeria: morphology and chronology. PLoS ONE.

[45] White, F. *The Vegetation of Africa: A Descriptive Memoir to Accompany the UNESCO/AETFAT/UNSO Vegetation Map of Africa* (UNESCO, 1983).

[46] Fick SE, Hijmans RJ (2017). WorldClim 2: new 1-km spatial resolution climate surfaces for global land areas. Int. J. Climatol..

[47] Diop, A. *Contribution à la Connaissance des Industries Paléolithiques Post-Acheuléennes dans la Presquîle du Cap-Vert* (Université Cheikh Anta Diop de Dakar, 1976).

[48] Bremond L, Alexandre A, Hély C, Guiot J (2005). A phytolith index as a proxy of tree cover density in tropical areas: valibration with leaf area index along a forest-savanna transect in southeastern Cameroon. Glob. Planet. Change.

[49] Bremond L (2008). Phytolith indices as proxies of grass subfamilies on East African tropical mountains. Glob. Planet. Change.

[50] Diester-Haass, L., Schrader, H.-J. & Thiede, J. Sedimentological and paleoclimatological investigations of two pelagic ooze cores off Cape barbas, North-West Africa. *Meteor Forschungsergebnisse: Reihe C, Geologie und Geophysik* **16**, 19–66 (1973).

[51] Dupont LM, Agwu COC (1992). Latitudinal shifts of forest and savanna in N. W. Africa during the Brunhes chron: further marine palynological results from site M 16415 (9° N 19° W). Veg. Hist. Archaeobot..

[52] Lezine AM, Casanova J (1991). Correlated oceanic and continental records demonstrate past climate and hydrology of North Africa (0–140 ka). Geology.

[53] Ramos J (2008). The Benzú rockshelter: a Middle Palaeolithic site on the North African coast. Quat. Sci. Rev..

[54] Jacobs Z (2017). The chronostratigraphy of the Haua Fteah cave (Cyrenaica, northeast Libya)—optical dating of early human occupation during Marine Isotope Stages 4, 5 and 6. J. Hum. Evol..

[55] *ROCEEH Database (ROAD). Locality Haua Fteah* https://www.roceeh.uni-tuebingen.de/roadweb (2021).

[56] *ROCEEH Database (ROAD). Locality Ifri n Ammar* https://www.roceeh.uni-tuebingen.de/roadweb (2021).

[57] Doerschner N (2016). A new chronology for Rhafas, northeast Morocco, spanning the North African Middle Stone Age through to the Neolithic. PLoS ONE.

[58] *ROCEEH Database (ROAD). Locality Rhafas Cave* https://www.roceeh.uni-tuebingen.de/roadweb (2021).

[59] Clark JD (2003). Stratigraphic, chronological and behavioural contexts of Pleistocene *Homo sapiens* from Middle Awash, Ethiopia. Nature.

[60] Slater, P. A. *Change in lithic technological organization strategies during the Middle and Later Stone Ages in east Africa*. Ph.D dissertation, Univ. of Illinois at Urbana-Champaign (2016).

[61] Backwell LR (2018). New excavations at Border Cave, KwaZulu-Natal, South Africa. J. Field Archaeol..

[62] Kuman K, Inbar M (1999). Palaeoenvironments and cultural sequence of the Florisbad Middle Stone Age hominid site, South Africa. J. Archaeol. Sci..

[63] Thompson E, Williams HM, Minichillo T (2010). Middle and Late Pleistocene Middle Stone Age lithic technology from Pinnacle Point 13B (Mossel Bay, Western Cape Province, South Africa). J. Hum. Evol..

[64] Marean CW (2010). The stratigraphy of the Middle Stone Age sediments at Pinnacle Point Cave 13B (Mossel Bay, Western Cape Province, South Africa). J. Hum. Evol..

[65] Backwell LR (2014). Multiproxy record of late Quaternary climate change and Middle Stone Age human occupation at Wonderkrater, South Africa. Quat. Sci. Rev..

[66] Scerri EML, Blinkhorn J, Niang K, Bateman MD, Groucutt HS (2017). Persistence of Middle Stone Age technology to the Pleistocene/Holocene transition supports a complex hominin evolutionary scenario in West Africa. J. Archaeol. Sci. Reports.

[67] Baluh, A. K. *The Middle Stone Age in West Africa: Lithics from the Birimi Site in Northern Ghana*. MSc thesis, Univ. of South Carolina (2017).

[68] Blinkhorn J, Timbrell L, Grove M, Scerri EML (2022). Evaluating refugia in recent human evolution in Africa. Philos. Trans. R. Soc. London, Ser. B.

[69] Timbrell L, Grove M, Manica A, Rucina S, Blinkhorn J (2022). A spatiotemporally explicit paleoenvironmental framework for the Middle Stone Age of eastern Africa. Sci. Rep..

[70] Durvasula A, Sankararaman S (2020). Recovering signals of ghost archaic introgression in African populations. Sci. Adv..

[71] Kaboth-Bahr S (2021). Paleo-ENSO influence on African environments and early modern humans. Proc. Natl Acad. Sci. USA.

[72] Shennan SJ, Crema ER, Kerig T (2015). Isolation-by-distance, homophily, and ‘core’ vs. ‘package’ cultural evolution models in Neolithic Europe. Evol. Hum. Behav..

[73] Shennan S (2001). Demography and cultural innovation: a model and its implications for the emergence of modern human culture. Cambridge Archaeol. J..

[74] Lycett SJ, Norton CJ (2010). A demographic model for Palaeolithic technological evolution: the case of East Asia and the Movius Line. Quat. Int..

[75] Will M, Kandel AW, Conard NJ (2019). Midden or molehill: the role of coastal adaptations in human evolution and dispersal. J. World Prehist..

[76] Lézine A (1997). Evolution of the West African mangrove during the Late Quaternary: a review. Geogr. Phys. Quat..

[77] Erlandson JM, Braje TJ (2015). Coasting out of Africa: the potential of mangrove forests and marine habitats to facilitate human coastal expansion via the southern dispersal route. Quat. Int..

[78] Guerin G, Mercier N, Adamiec G (2011). Dose-rate conversion factors: update. Ancient TL.

[79] Prescott JR, Hutton JT (1994). Cosmic ray contributions to dose rates for luminescence and ESR dating: large depths and long-term time variations. Radiat. Meas..

[80] Aitken M (1989). Luminescence dating: a guide for non-specialists. Archaeometry.

[81] Nathan RP, Mauz B (2008). On the dose-rate estimate of carbonate-rich sediments for trapped charge dating. Radiat. Meas..

[82] Bateman MD, Catt JA (1996). An absolute chronology for the raised beach and associated deposits at Sewerby, East Yorkshire, England. J. Quat. Sci..

[83] Murray AS, Wintle AG (2003). The single aliquot regenerative dose protocol: potential for improvements in reliability. Radiat. Meas..

[84] Bateman MD, Frederick CD, Jaiswal MK, Singhvi AK (2003). Investigations into the potential effects of pedoturbation on luminescence dating. Quat. Sci. Rev..

[85] Galbraith RF, Roberts RG, Laslett GM, Yoshida H, Olley JM (1999). Optical dating of single and multiple grains of quartz from jinmium rock shelter, northern australia: part I, experimental design and statistical models. Archaeometry.

[86] Neumann K (2019). International code for phytolith nomenclature (ICPN) 2.0. Ann. Bot..

[87] Blinkhorn, J. & Grove, M. Explanations of variability in Middle Stone Age stone tool assemblage composition and raw material use in Eastern Africa. *Archaeol. Anthropol. Sci*. **13**, 14 (2021).

[88] Spratt RM, Lisiecki LE (2016). A Late Pleistocene sea level stack. Clim.

[89] Spalding, M., Kainuma, M. & Collins, L. World atlas of mangroves (version 3.1). ITTO, ISME, FAO, UNEP-WCMC, UNESCO-MAB, UNU-INWEH & TNC 10.34892/w2ew-m835 (2010).

[90] GEBCO Bathymetric Compilation Group. The GEBCO_2019 Grid - a continuous terrain model of the global oceans and land. British Oceanographic Data Centre, National Oceanography Centre, NERC, UK; 10.5285/836f016a-33be-6ddc-e053-6c86abc0788e (2019).

[91] Dinerstein E (2017). An ecoregion-Bbased approach to protecting half the terrestrial realm. Bioscience.

[92] Olson DM (2001). Terrestrial ecoregions of the world: a new map of life on Earth. Bioscience.

[93] Laskar J (2004). A long-term numerical solution for the insolation quantities of the Earth. Astron. Astrophys..

[94] Lisiecki, L. E. & Raymo, M. E. Diachronous benthic δ^18^O responses during Late Pleistocene terminations. *Paleoceanography* **24**, PA3210 (2009).

[95] Castañeda IS (2009). Wet phases in the Sahara/Sahel region and human migration patterns in North Africa. Proc. Natl Acad. Sci. USA.

[96] Krapp, M., Beyer, R., Edmundson, S., Valdes, P. & Manica, A. A statistics-based reconstruction of high-resolution global terrestrial climate for the last 800,000 years. *Sci. Data* **8**, 228 (2021) 10.1038/s41597-021-01009-3 PMC8397735 34453060

[97] Beyer R, Krapp M, Manica A (2020). An empirical evaluation of bias correction methods for palaeoclimate simulations. Clim.

