# Supplementary material for: Longstanding behavioural stability in West Africa extends to the Middle Pleistocene at Bargny, coastal Senegal
Source: Nat Ecol Evol. 2023 May 4;7(7):1141–51. doi: 10.1038/s41559-023-02046-4 (PMC10333124; doi:10.1038/s41559-023-02046-4)
Supplement: Supplementary file 1 — Supplementary Information 1: luminescence dating (including Supplementary Tables 1.1–1.2 and Figs. 1.1–1.3). Supplementary Information 2: plant microfossils (including Supplementary Tables 2.1–2.3 and Figs. 2.1–2.18). Supplementary Information 3: archaeological comparisons and refugia analysis (including Supplementary Tables 3.1–3.2 and Fig. 3.1). [file 41559_2023_2046_MOESM1_ESM.pdf]

nature ecology & evolution

Article https://doi.org/10.1038/s41559-023-02046-4

Longstanding behavioural stability in West Africa extends to the Middle Pleistocene at Bargny, coastal Senegal

In the format provided by the authors and unedited

Longstanding behavioural stability in West Africa extends to the Middle Pleistocene at Bargny, coastal Senegal

Niang, Blinkhorn, Bateman & Kiahtipes

Supplementary Information 1: Luminescence Dating

1. Introduction

A total of five samples from the Bargny 1, Senegal underwent luminescence dating the Sheffield Luminescence Laboratory.

Naturally occurring potassium (K), thorium (Th), uranium (U) are the main contributors of dose to sedimentary quartz. The concentrations of these elements in the sampled sandy sediments (excluding limestone clasts) were determined by inductively coupled plasma mass spectrometry (ICP) at SGS laboratories Ontario Canada (SI Table 1.1). Elemental concentrations were converted to annual dose rates using data from Guerin and colleagues[1]. Calculations took into account attenuation factors relating to sediment grain sizes used, density and palaeomoisture (SI Table 1.1). Attenuation of dose by moisture used present-day values with a ± 3 % error to incorporate fluctuations through time (SI Table 1.1). Contributions of gamma-dose from adjacent units was modelled using data from Aitken[2]. The contribution to dose rates from cosmic sources was calculated using the expression published in Prescott and Hutton[3] (SI Table 1.1).

SI Table 1.1: Summary of dosimetry related data.

Lab Code	U (PPM)	Th (PPM)	K (%)	D_{cosmic} (μGy/a^{-1})	Moisture (%)	Dose rate *** (μGy/a^{-1})
Shfd19079	1.41	6.9	0.05**	188 ± 9	3.4	1045 ± 40
Shfd19080	2.15	5.8	0.1	154 ± 8	4.9	1135 ± 45
Shfd19081	1.59	3.5	0.05**	140 ± 7	2.7	813 ± 32
Shfd19082	1.92	4.1	0.05**	137 ± 7	2.5	881 ± 36
Shfd19083	1.45	4.5	0.05**	132 ± 7	2.5	840 ± 33

** K below ICP detection limits of 0.1% so 0.05% assumed.
*** Dose rates adjusted for gamma dose received from adjacent units using data from Aitken (1989).

As calculated the calculated dose rates are based on analyses of the sediment sampled at the present day. This assumption is only valid if no movement and/or re-precipitation of the four key elements has taken place since sediment burial. The impacts of possible post-depositional changes to carbonate content (See Nathan and Mauz[6]) was considered. We undertook dose rate analysis both based on sediments as they were sampled and on the sediments once thy had been stripped of carbonate using concentrate HCL. This allowed three models to be developed. Firstly a model where carbonate deposition was at a similar time to sediment deposition. Secondly a linear uptake model of carbonate through burial time and thirdly a model with recent (modern) carbonate deposition. For sample Shfd19082 this gave ages of (1) 93±4 ka, (2) 108±5 ka and (3) 117±6 ka for the respective models. Despite changing the dose rate by up to 30%, no model resolved the age inversion seen within Unit 4 between samples Shfd19081 (134±5 ka) and Shfd19082 (93±4 ka). Whilst it might be tempting to report the results of model 3 for Shfd19082, thereby minimising the age reversal, having looked in detail at the sediments, whilst the % carbonate is relatively high for this sample, it is in clast form indicative of erosion and deposition of local limestone rather than post-depositional pedogenic or groundwater

calcrete carbonates. Thus we had no reason to go with either model 2 or 3 and ages are reported based on the dose rate of model 1. The presence of large limestone clasts within Unit 4 could have impacted on dose-rate in another way. As dose-rates were based on ICP analysis of bulk sediment an infinity field was assumed. Dose hot/cold spots from large clasts not included in the bulk sample and ICP analysis could have affected gamma dose rates for Unit 4. Whilst in situ field measurements of gamma dose would have incorporated this aspect into any dose-rate calculations, such measurements were logistically impossible at the time of sampling. Whilst unquantified, this is unlikely to have caused a the >40% change in dose-rate required to resolve the age reversal between samples Shfd19081 and Shfd19082. Future in-situ gamma-dose measurements might resolve whether the age reversal is in part due to dose-rate over-estimation from the unsampled clastic material. Future higher resolution dating sampling and Bayesian modelling of ages might also help resolve the age reversal and help understand its cause.

The luminescence samples were prepared under subdued red lighting following the procedure to extract and clean quartz outlined in Bateman and Catt[4]. Material for dating was taken from prepared quartz isolated to a size range of 90-180 µm as the original sediment was fined grained. As this size range is sub-optimal for single grain measurement, initial measurements were made at the single aliquot level. For this, grains were mounted as a 8 mm diameter monolayer on 9.6 mm diameter stainless still disks using silkospray. The samples underwent measurement using a Risø DA-20 luminescence reader with radiation doses administered using a calibrated $_{90}$strontium beta source. Stimulation was with blue/green LEDs and luminescence detection was through a Hoya U- 340 filter. Samples were analysed using the single aliquot regenerative (SAR) approach[5,6], in which an interpolative growth curve is constructed using data derived from repeated measurements of a single aliquot which has been given various laboratory irradiations (Figure 1.1b). Five regeneration points were used to characterise growth curves, with the first regeneration point being identical to the last in order to check if sensitivity changes caused by repeated measurement of the same grains are correctly monitored and corrected for by the SAR protocol (known as the “recycling ratio”). The most appropriate preheat temperature for the samples was selected using a dose recovery preheat plateau test (Figure S1.2). This resulted in selection of preheat temperatures of 260 °C for 10 seconds which was applied to prior to each OSL measurement to remove unstable signal generated by laboratory irradiation. De values from individual aliquots were only accepted if they exhibited an OSL signal measurable above background, good growth with dose, recycling values within ± 10 % of unity, and the error on the test dose used within the SAR protocol was less than 20 %. The samples possessed good luminescence characteristics with a rapid decay of OSL with stimulation and OSL signals dominated by a fast component (e.g. Figure S1.1a). Within the SAR protocol results which grew well with laboratory dose (e.g. Figure 1.1b).

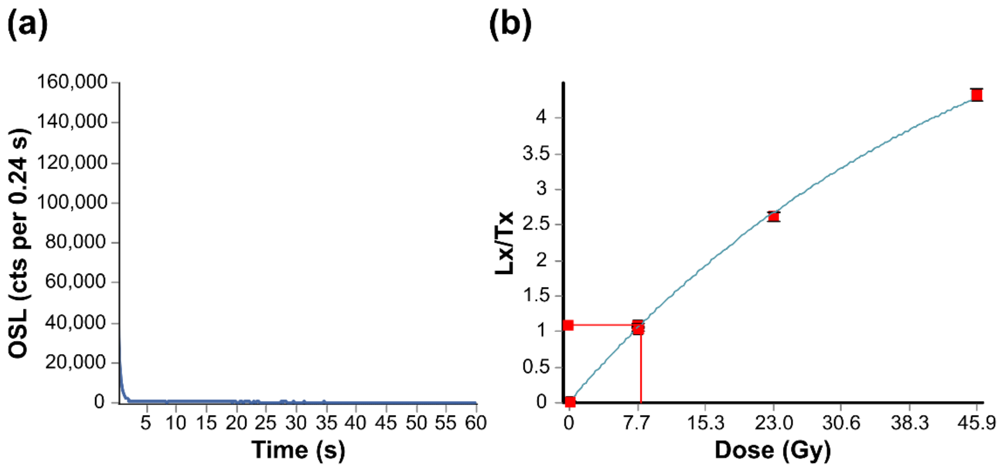

SI Figure 1.1: Example of single aliquot OSL data for sample Shfd19079: (a) OSL decay of naturally acquired signal; (b) SAR growth curve The red line represents interpolation of the natural dose (De); Error bars on Sar growth curve based on counting statistics

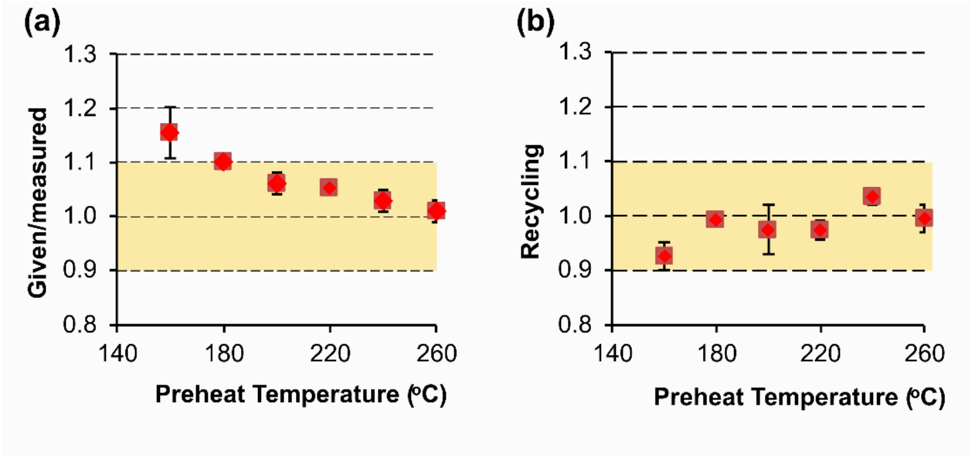

SI Figure 1.2: Results of different preheat temperatures (Data presented as mean with error bars based on n=3 per temperature) in recovering a ~20 Gy beta radiation dose from sample Shfd19081 (a) Given to recovered dose ratio at different preheat temperatures. (b) recycling ratio (ratio between the first and last dose point) at the different preheat temperatures. Data points in both plots are the averages of three measurements performed for each preheat temperature. Yellow shading indicates acceptable limits for dose recovery and recycling.

The effects of incomplete bleaching of the sediment during the last period of transport or exposure in situ can be profound. Typically, poorly bleached sediments retain a significant level of residual signal from previous phases of sedimentary cycling, leading to inherent inaccuracies in the calculation of a palaeodose value. By plotting the replicate De data for the samples (Figure 3) some assessment of whether older or younger material has been included in the sample measurements can be made. In principle a well-bleached sample that has not been subjected to post-depositional disturbance should have replicate De data which is normally distributed and highly reproducible (see [7]: Figure S1.3; [8]). Where post-depositional disturbance or incomplete bleaching prior to sample burial has occurred skewing of this distribution may occur and/or replicate reproducibility may be lower[8,9]. In the case of

poorly bleached material skewing should be evident with a high De tail[10]. High De tails may also be indicative of saturated samples and interpolation of the De values from the upper, low gradient part of the growth curve[11].

As Figure S1.3 demonstrates, the De replicate distributions for the samples are normally distributed (especially after outliers are removed) with low levels of De replicate scatter (overdispersion or OD <25%; values given in SI Table 1.2). This data shows no indication that either partial bleaching or post-depositional disturbance. As a consequence De values for age calculation purposes have been extracted using the Central Age Model (CAM) of Galbraith and Green[12].

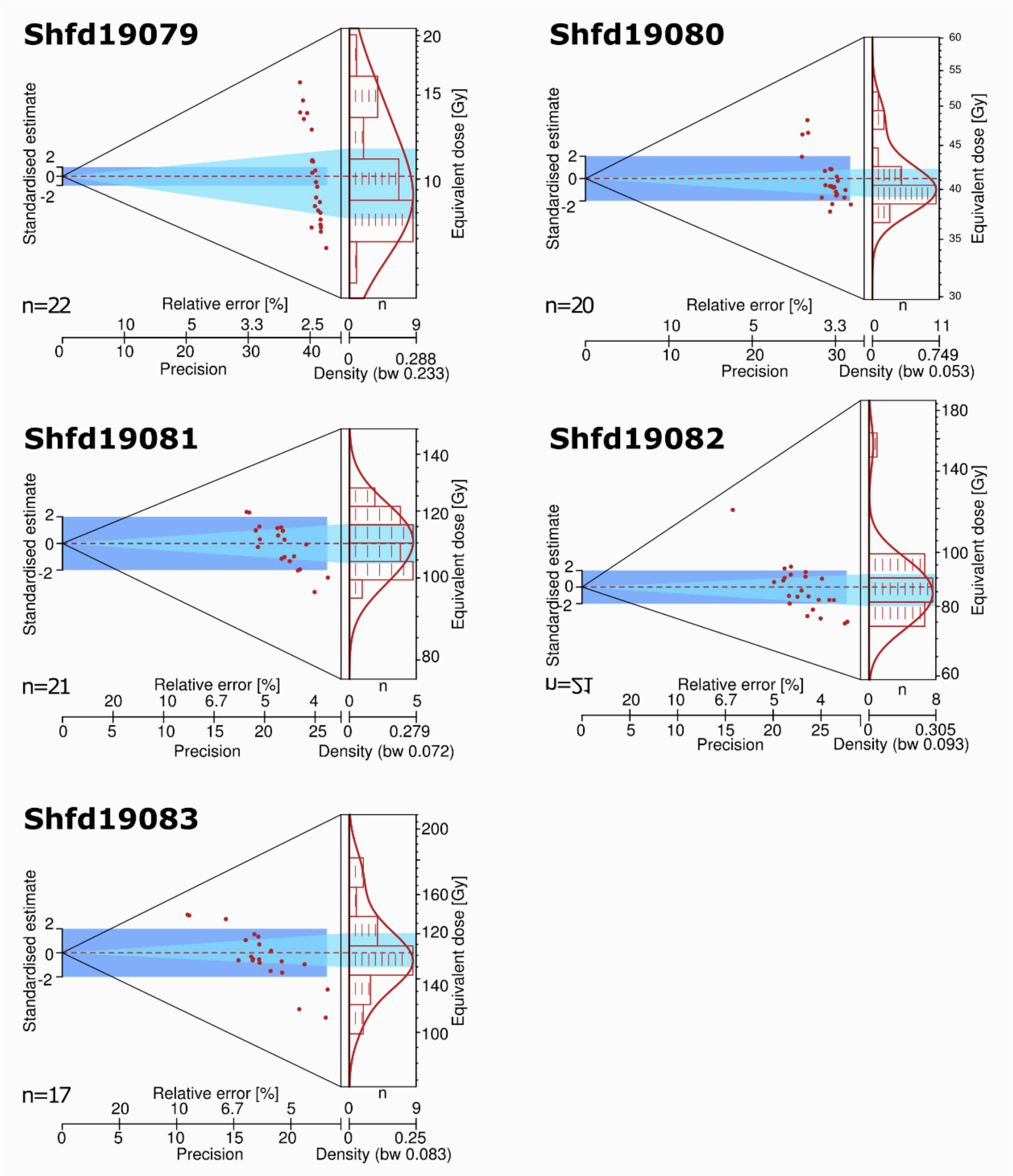

SI Figure 1.3: Abanico De distribution plots for OSL samples collected from Bargny, Senegal showing normal distribution, low overdispersion and considered indicative of sediment well bleached at burial and not post-depositionally disturbed.

SI Table 1.2: Selected elemental data as measured by ICP-MS where Unit 4 samples (Shfd 19082) show distinct differences with over and underlying units as well as high variability within Unit 4.

Sample	Al %	Ca %	Cr ppm	Fe %	Mn ppm	Ni ppm	Si %	Sr ppm	Ti %	Zn ppm	Co ppm	Cs ppm	Nb ppm	Rb Ppm
Shfd 19079	3.46	0.7	236	1.95	138	700	>30	60	0.37	33	5.3	1.7	10	10.7
Shfd 19080	3.61	6.2	243	1.97	138	747	>30	109	0.32	30	4.9	1.5	10	12.8
Shfd 19081	2.13	20.6	134	1.36	482	327	18.2	179	0.20	24	8.6	0.6	6	4.9
Shfd 19082	2.23	15.3	169	1.84	509	488	24.7	151	0.24	25	10.4	0.8	7	6.6
Shfd 19083	2.74	5.2	189	1.75	141	408	>30	101	0.29	29	6.2	1.0	9	8.1

Ages are quoted in years from the present day (2019) and are presented with one sigma confidence intervals which incorporate systematic uncertainties with the dosimetry data, uncertainties with the palaeomoisture content and errors associated with the De determination. Data shows that the samples had high reproducibility in terms of their palaeodose and therefore ages should reflect true burial ages.

Supplementary Information 2: Plant Microfossils

1. Summary

This supplement describes the laboratory preparation, analysis, and quantitative analysis of plant microfossils recovered from the Bargny 1 deposits. In the field, samples were collected at 5 cm intervals across the entire profile. Because the deposits do not exhibit a uniform age-depth profile and analysis of phytoliths/pollen is labor intensive, subsampling efforts focused on evaluating changes across the MSA archaeological strata in detail (Units 4-6) and general characterization of the major units making up the overlying deposits (Units 1-3). The 31 samples processed and analyzed for plant microfossils are listed in SI Table 2.1.

2. Laboratory methods

Sediment samples were ground, passed through a 250-micron sieve, and placed in a shaker overnight with Calgon solution (sodium hexametaphosphate) before having sands and clays separated by settling and centrifugation-decant cycles at 277 g for 2 minutes. At this point, samples were spiked with Lycopodium spores and treated with 10% HCl in a 40º bath for 10 minutes. After centrifugation-decant cycles until pH neutral, the samples were separated by density using a solution of zinc bromide and 5% HCl with a specific gravity of 2.3 g/ml. The resulting residue was extracted in ethanol and transferred to glycerol for analysis.

This set of laboratory methods for extracting phytoliths is based on published methods for digesting terrestrial sediments and recovering pollen and phytolith microfossils[13,14], but omits the use of a strong oxidizing agent such as peroxide or nitric acid. Judging from the well-oxidized state of the sediments, further degradation of the organic remains would only remove valuable information represented by organic microfossils (pollen microfossils, amoebas, etc.), but also permits the use of Lycopodium spores to track laboratory errors and to calculate microfossil concentrations.

SI Table 2.1: Samples, weights, tracer spores, diagnostic phytoliths, total phytoliths

SAMPLE	depth	stratum	wt_gm	LYCO_n	TOTAL_diag	TOTAL_Phyt
BG1.020	20	Unit 1	5.06	239	151	382
BG1.040	40	Unit 2	6.04	212	64	123
BG1.060	60	Unit 2	6.05	57	227	464
BG1.090	90	Unit 2	5.35	100	16	23
BG1.100	100	Unit 3	6.02	200	29	70
BG1.110	110	Unit 3	5.2	360	203	398
BG1.150	150	Unit 3	6	423	256	548
BG1.175	175	Unit 3	6.27	165	210	370
BG1.195	195	Unit 3	6.02	344	208	389
BG1.210	210	Unit 3	5.06	20	215	510
BG1.220	220	Unit 4	6.02	134	223	460
BG1.230	230	Unit 4	6.03	244	67	130
BG1.240	240	Unit 4	6	287	200	385
BG1.255	255	Unit 5	6.05	163	374	696
BG1.270	270	Unit 5	6.03	20	226	468
BG1.280	280	Unit 5	6.02	11	231	364
BG1.290	290	Unit 5	6.02	66	301	648
BG1.295	295	Unit 5	6.27	78	231	519
BG1.300	300	Unit 5	5.04	214	290	561
BG1.305	305	Unit 5	5.03	309	213	374
BG1.310	310	Unit 5	6	258	21	34
BG1.315	315	Unit 5	6.01	162	196	355
BG1.320	320	Unit 6	6.05	114	236	461
BG1.325	325	Unit 6	6.01	370	206	435
BG1.330	330	Unit 6	6.01	119	203	459
BG1.335	335	Unit 6	6.02	465	277	569
BG1.340	340	Unit 6	6.02	49	216	472
BG1.345	345	Unit 6	6.03	44	39	75
BG1.350	350	Unit 6	6.06	0	13	28
BG1.355	355	Unit 6	6	3	214	478
BG1.360	360	Unit 6	6	400	207	401

3. Microfossil identification

3.1 Phytolith Types and Images

Phytolith and pollen microfossils were identified using a binocular light microscope at 400x-1000x magnification. Phytolith nomenclature and categories follow the International Code for Phytolith Nomenclature[15] , but we tried specifically to create sample categories consistent with Bremond and colleagues[16,17] assessment of phytoliths from surface samples across West Africa (Table 2). Samples were analyzed until at least 200 diagnostic phytoliths (Figures) were encountered (SI Table 2.2) or until 300 Lycopodium tracers were encountered. Non-diagnostic phytolith counts were tracked, but are not considered in the phytolith sum.

SI Table 2.2: Phytolith classifications from ICPT[15] compared to Bremond and colleagues[16]

AFFILIATION	TYPE	GROUP	Bremond_et_al_2005	ICPN 2.0
DICOTYLEDONS	SPH_ORN	WOODY PLANTS	Rough spherical	Sphereoid Ornate
DICOTYLEDONS	SPH_CMP	WOODY PLANTS	Non-diagnostic	Not Listed
ARECACEAE	SPH_ECH	PALMS	Crenate spherical	Sphereoid Echinate
MONOCOTYLEDONS*	ACU_BUL	MONOCOTS	Point-shaped	Acute Bulbosus
POACEAE_CYPERACEAE	BUL_FLA	MONOCOTS	Fan-shaped	Bulliform Flabellate
POACEAE	PAP	GRASSES	Cone-shaped	Papillate
POACEAE	RON	GRASSES	Non-diagnostic	Rondel
POACEAE	BIL	GRASSES	Dumbell	Bilobate
POACEAE	CRO	GRASSES_PANICOID	Cross	Cross
POACEAE	SAD	GRASSES_CHLORIDOID	Saddle	Saddle
POACEAE*	ELO_SIN	ELONGATE_GRASSES	Non-diagnostic	Elongate Sinuate
POACEAE*	ELO_DET	ELONGATE_GRASSES	Non-diagnostic	Elongate Dentate
POACEAE*	ELO_DEN	ELONGATE_GRASSES	Non-diagnostic	Elongate Dendritic
VARIOUS	SPH_PSI	UNDIFF	Smooth spherical	Sphereoid Psilate
VARIOUS	ELO_ENT	UNDIFF	Non-diagnostic	Elongate Entire
VARIOUS	TRA_ANN	UNDIFF	NA	Tracheary Annulate/Helical
VARIOUS	TRA_PIT	UNDIFF	NA	Tracheary Pitted
VARIOUS	TRA_BOR	UNDIFF	NA	Tracheary Bordered
VARIOUS	BLO	UNDIFF	Fan-shaped	Blocky
VARIOUS	UNDIFF	CORE_DETAILS	Non-diagnostic	Not Listed
CHARRED_DIAGNOSTIC	CHARRED	CORE_DETAILS	NA	NA

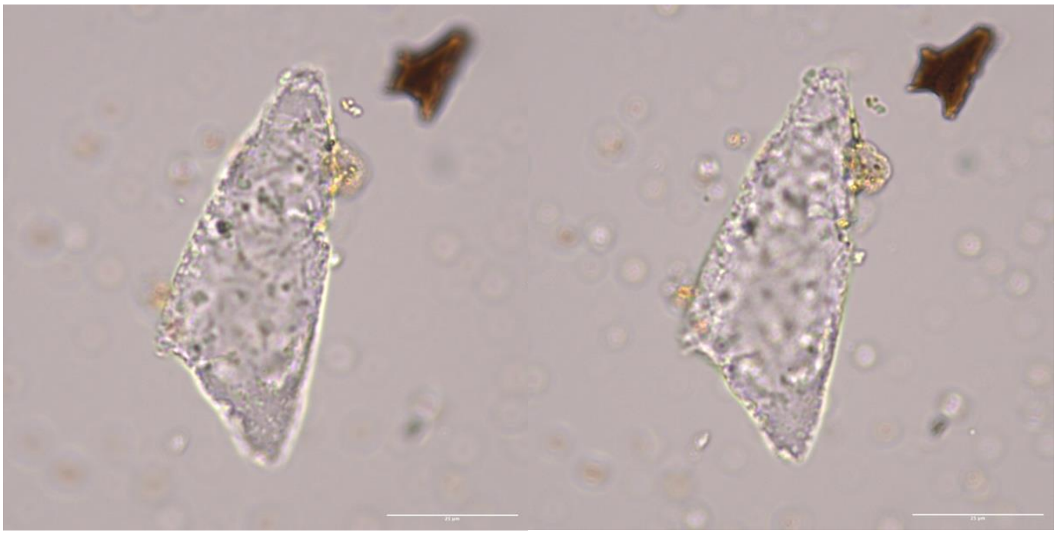

SI Figure 2.1: Acute Bulbosis (ACU-BUL) phytolith, scale = 25 microns**.**

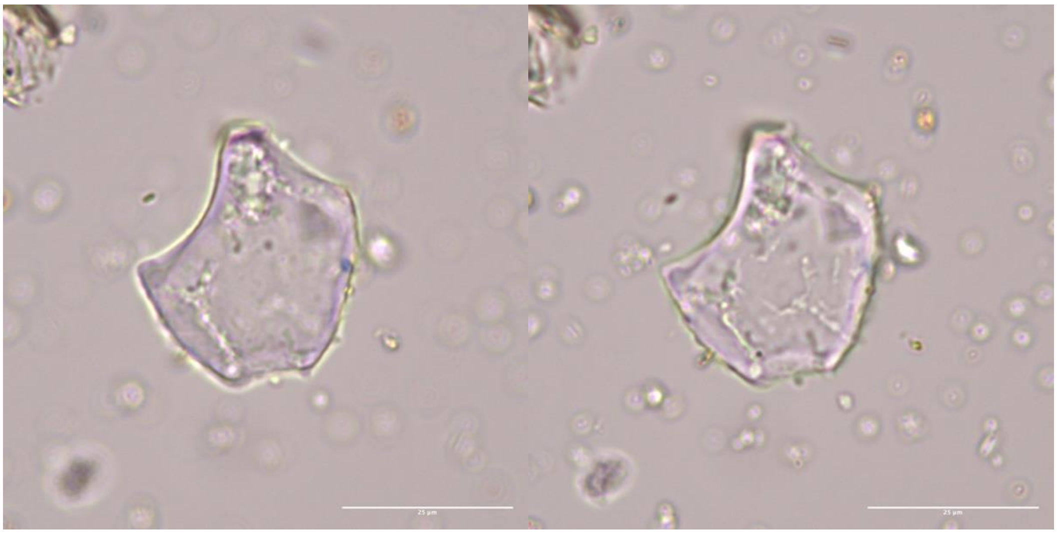

SI Figure 2.2: Flabellate (FLA) phytolith, scale = 25 microns.

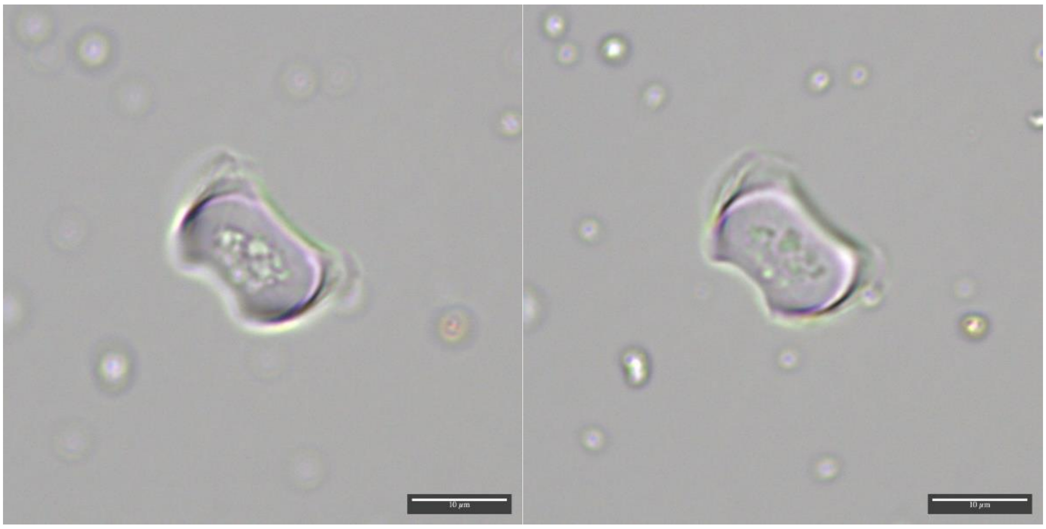

SI Figure 2.3: Saddle (SAD) phytolith, scale = 10 microns.

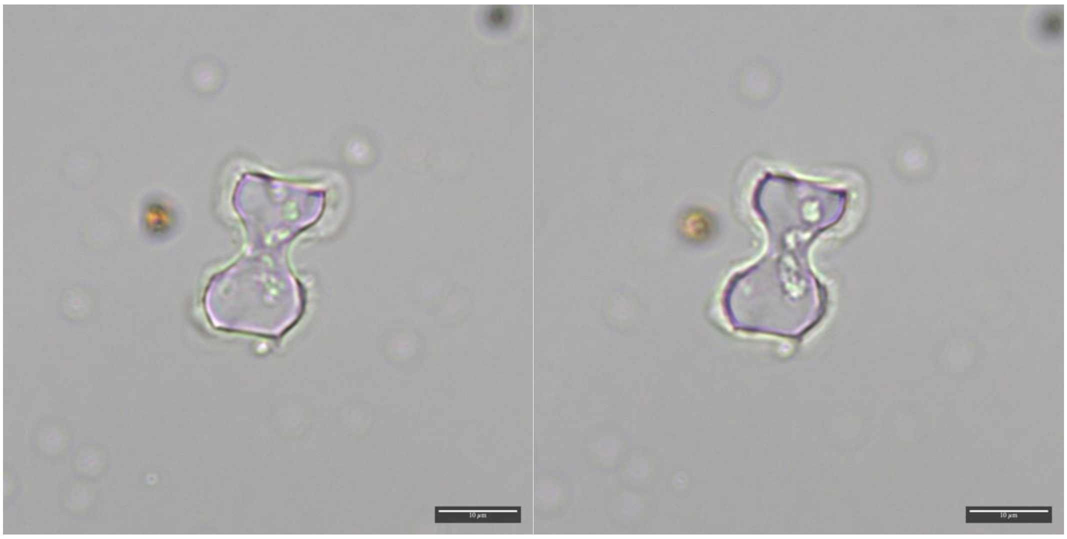

SI Figure 2.4: Bilobate (BIL) phytolith, scale = 10 microns.

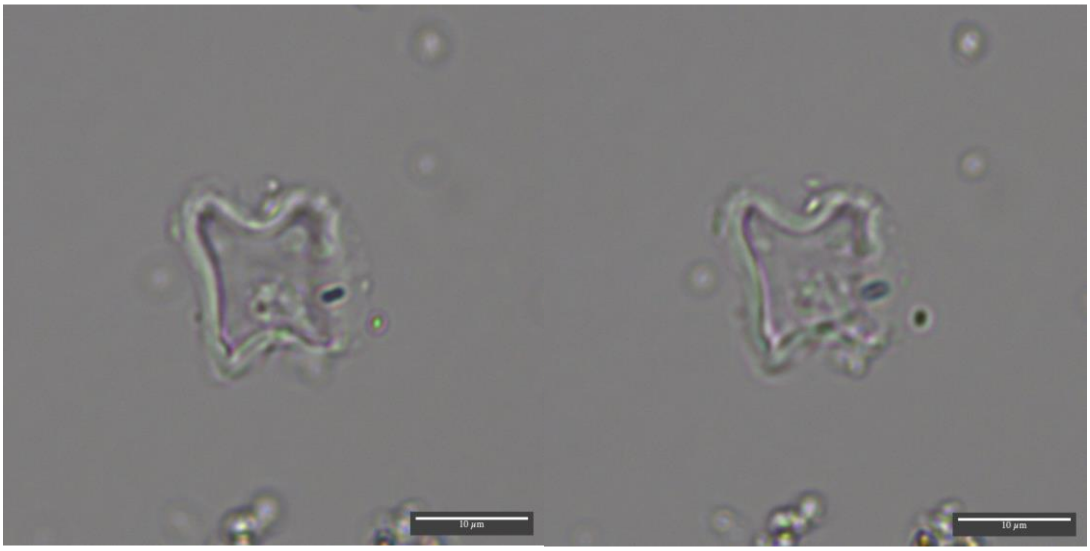

SI Figure 2.5: Cross-shaped (CRO) phytolith, scale = 10 microns.

3.2 Pollen Types and Images

SI Table 2.3: Fossil pollen types with family names, plotting groups, and labels used in diagrams.

FAMILY	TAXON		GROUP	LABEL
	CYPERACEAE	Cyperaceae undiff.	WETLAND_HERBS	Cyperaceae
TYPHACEAE		Typha	WETLAND_HERBS	Typha
	POACEAE	Poaceae undiff.	GRASSES	Poaceae
OLEACEAE		Olea-type	TRADE_WIND	Olea-type
PINACEAE		Pinaceae undiff.	TRADE_WIND	Pinaceae
AMARANTHACEAE		Amaranthaceae undiff.	XERIC	Amaranthaceae
ASTERACEAE		High-Spine Asteraceae	XERIC	Asteraceae-HS
ASTERACEAE		Low-Spine Asteraceae	XERIC	Asteraceae-LS
NYCTAGYNACEAE		Boerhavia	XERIC	Boerhavia
MYRTACEAE		Syzygium	RIPARIAN_TREES-SHRUBS	Syzygium
AVICENNIACEAE		Avicennia	RIPARIAN_TREES-SHRUBS	Avicennia
COMBRETACEAE-MELASTOMATACEAE		Combretaceae-Melastomataceae undiff.	SUDANEO-GUINEAN_TREES-SHRUBS	Comb.-Mel.
CONVOLVULACEAE		Ipomoea-type	SUDANEO-GUINEAN_TREES-SHRUBS	Ipomoea-type
CANNABACEAE		Celtis	SUDANEO-GUINEAN_TREES-SHRUBS	Celtis
CANNABACEAE		Trema orientalis	SUDANEO-GUINEAN_TREES-SHRUBS	Trema orientalis

FABACEAE-CAESALPINIOIDAE	Detarium	SUDANEO-GUINEAN_TREES-SHRUBS	Detarium
FABACEAE-MIMOSOIDAE	Acacia-type	SUDANEO-GUINEAN_TREES-SHRUBS	Acacia-type
MALVACEAE	Adansonia	SUDANEO-GUINEAN_TREES-SHRUBS	Adansonia
RUBIACEAE	Spermacoce-type	SUDANEO-GUINEAN_TREES-SHRUBS	Spermacoce-type
SAPINDACEAE	Dodonaea-type	SUDANEO-GUINEAN_TREES-SHRUBS	Dodonae-type

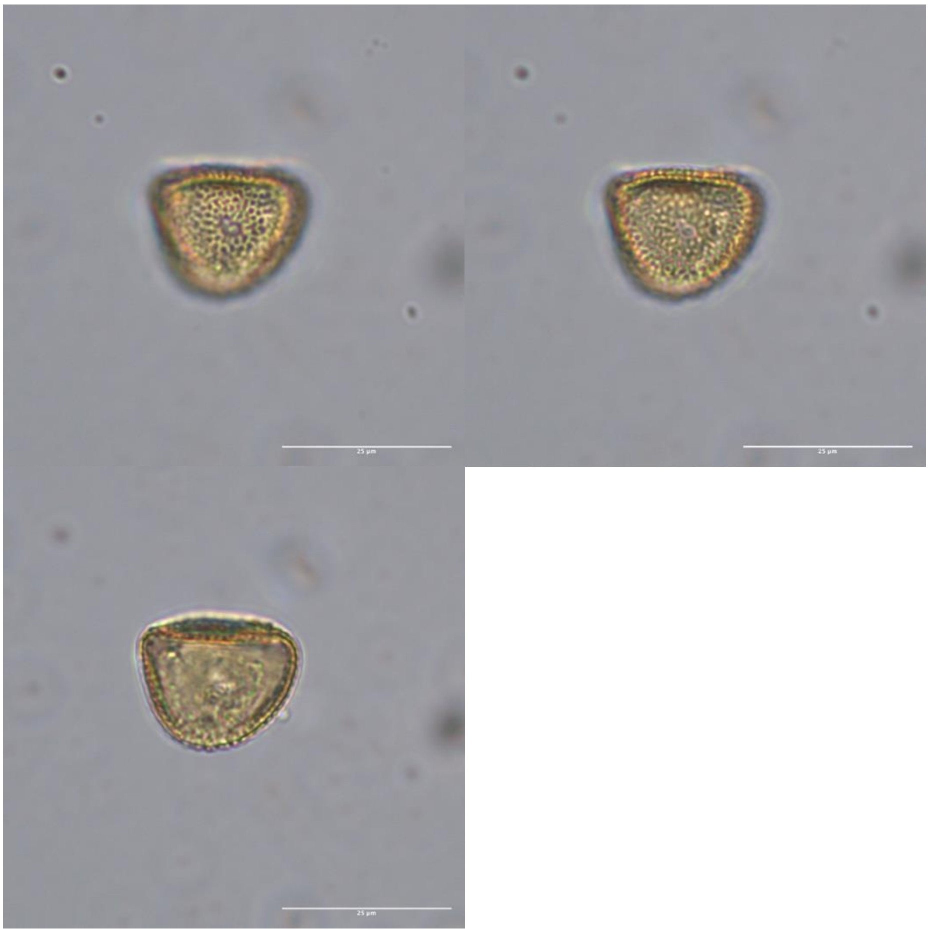

SI Figure 2.6: Typha pollen, scale = 25 microns

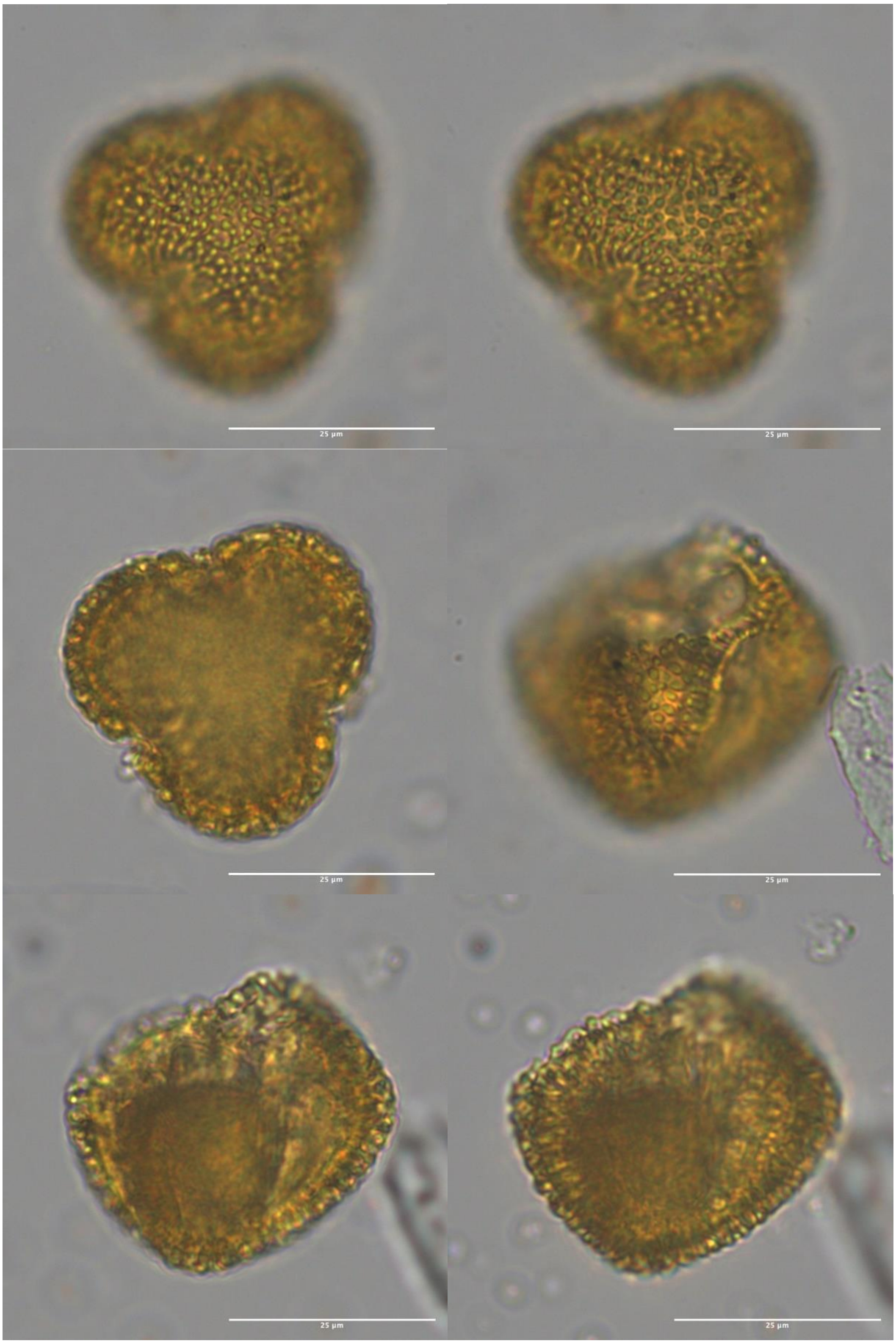

SI Figure 2.7 -Avicennia pollen, scale = 25 microns.

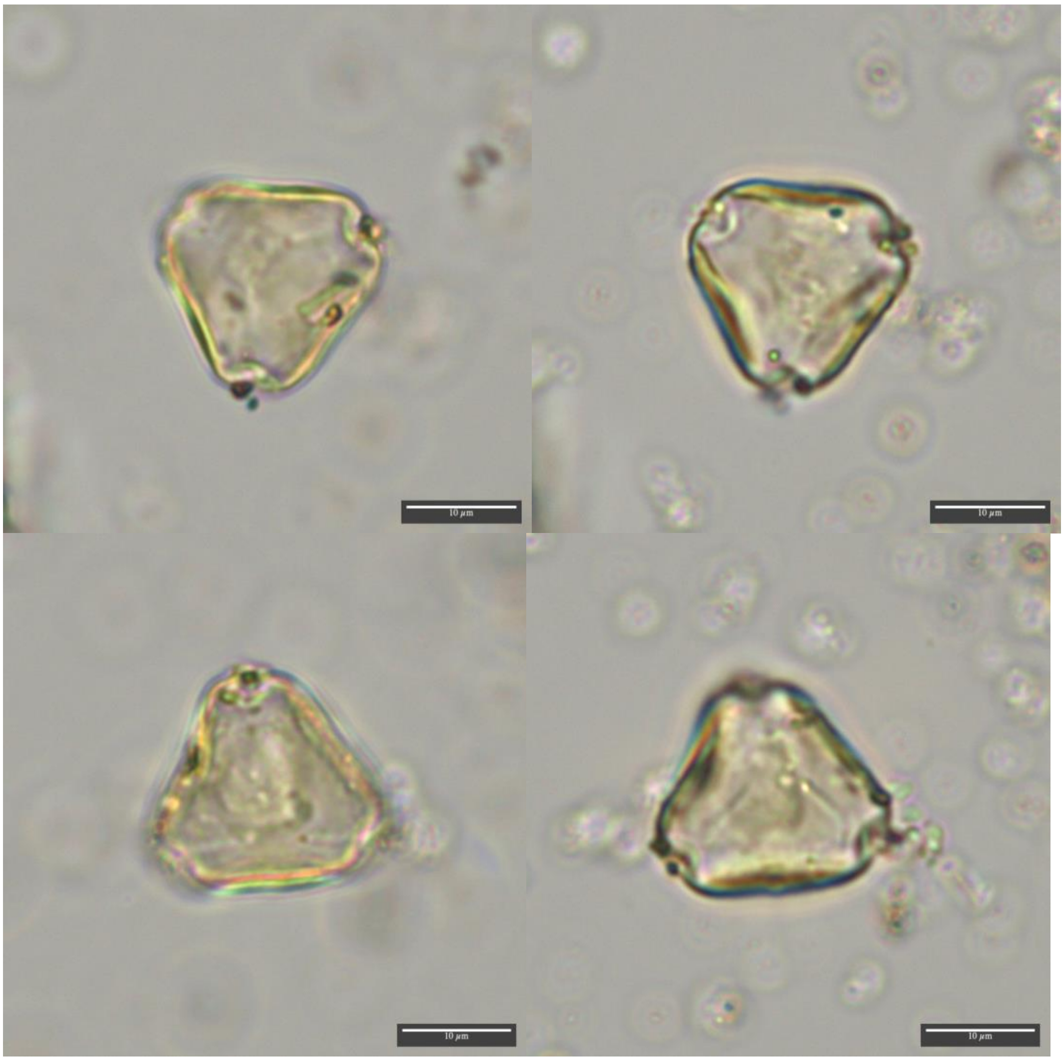

SI Figure 2.8 -Syzygium pollen, scale = 10 microns

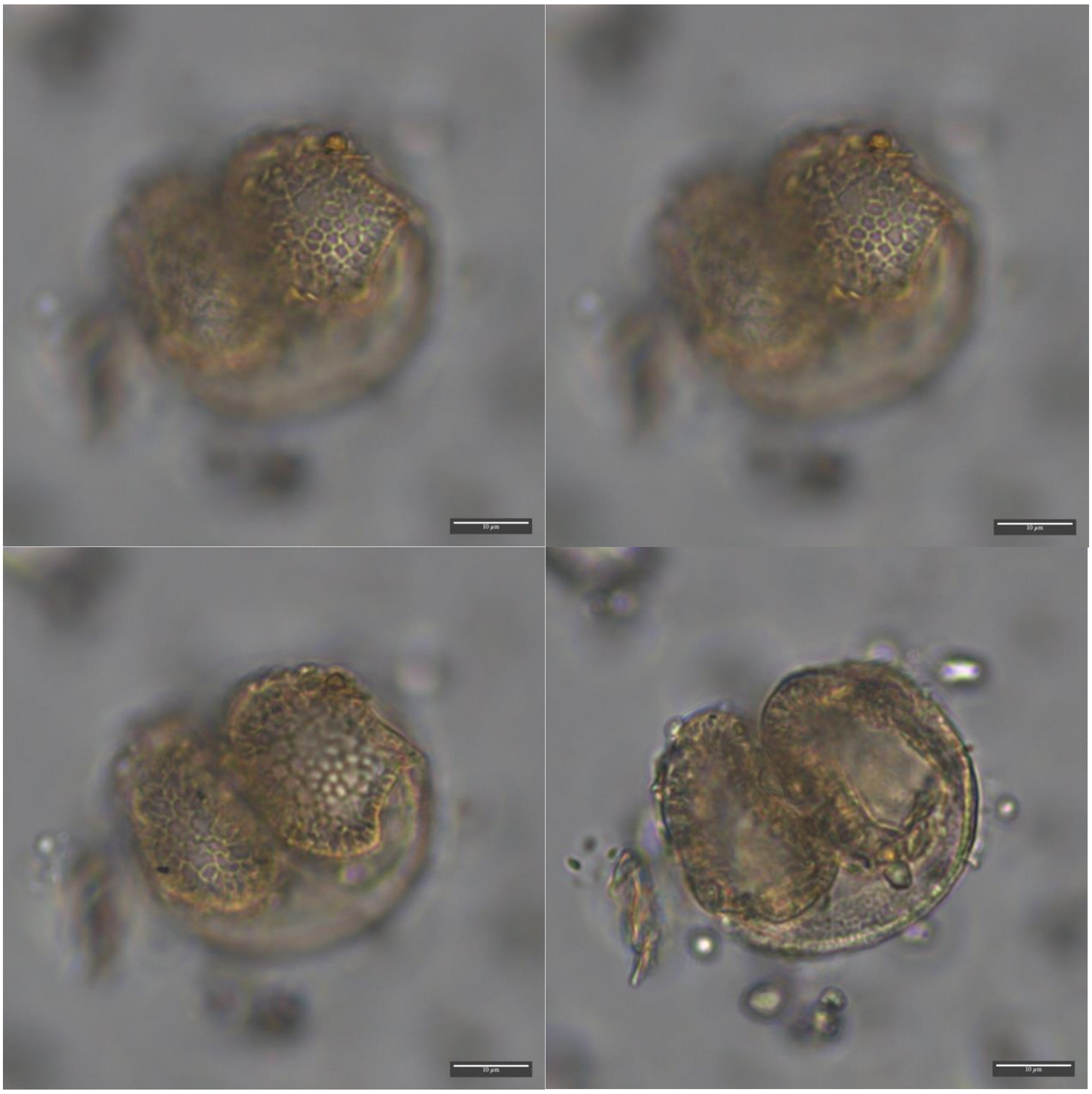

SI Figure 2.9 -Pinaceae pollen, scale = 10 microns.

4. Plant Microfossil Recovery, Preservation, and Concentration

Concentrations of phytoliths and pollen were established by tracking Lycopodium spores encountered during analysis. Samples where 200 Lycopodium were encountered before 50 identifiable phytoliths were considered unreliable. Although the number of diagnostic pollen sampled does reach 100 in some samples, the number of damaged, fragmented, and indeterminate types is too high to pursue reliable statistical analysis of the pollen data. In general, the patterns in microfossil deposition/preservation (SI Figure 2.10) closely follow the site's sedimentology, with the highest concentrations occurring in Units 3, 4, and 5 in tandem with higher values for organic matter and sediment sorting. The concentrations and preservation of plant microfossils signals a major change in the depositional environment between Unit 3 and Unit 4.

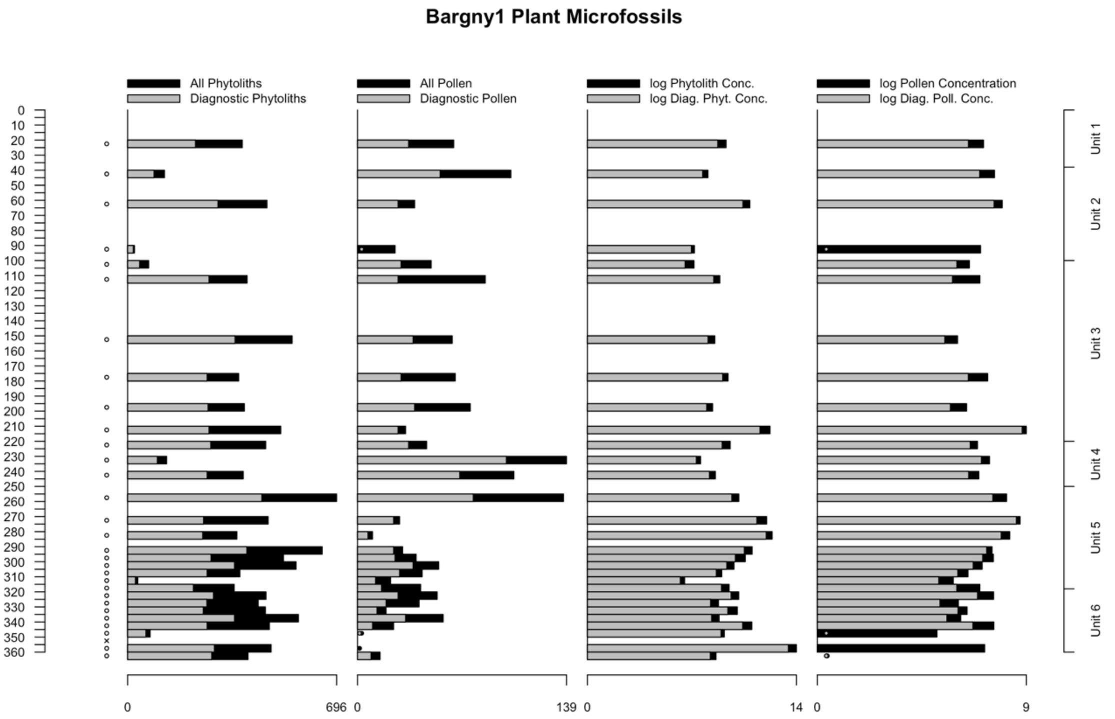

SI Figure 2.10: Barplots showing (L to R) counts of diagnostic (grey bars) and all (black bars) for phytoliths and pollen. The plots on the right half show log transformed concentration values (fossils/g) for phytoliths and pollen.

5. Phytolith Results

Phytoliths from the Bargny 1 samples show a strong representation of Chloridoideae and Panicoideae phytolith types (BIL and CRO, respectively) as well as types belonging to undifferentiated monocots (ACU_BUL, BUL_FLA, and RON) (SI Figure 2.11). The frequency of SPH_ECH phytoliths is high in some of the lowermost samples, but we must use caution in interpreting this morphotype as it overlaps with sponge spicules common in coastal zones[15]. While this type was tracked during analysis, this type is excluded from the statistical analyses. Samples with excessive SPH_PSI and SPH_ECH tend to be the most weathered and have the poorest preservation. These are excluded from further analysis.

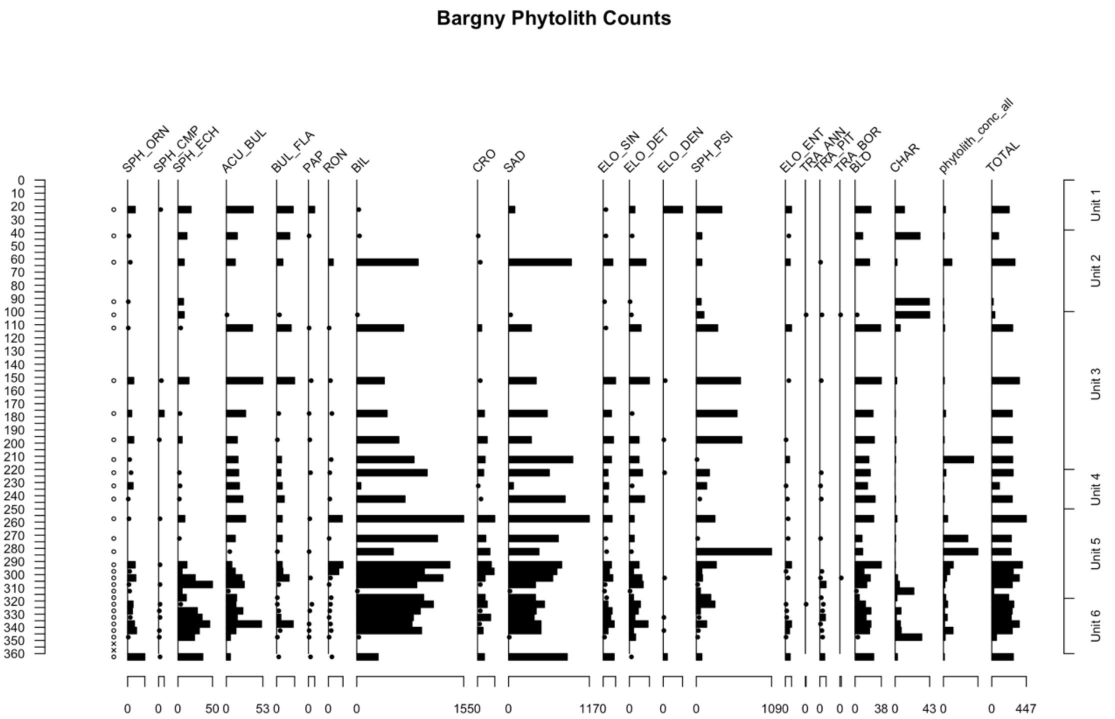

SI Figure 2.11: Phytolith results as number of identifications per sample.

6. Pollen Results

Identifiable pollen was preserved in the samples and provides some insights into the kinds of vegetation cover that grew at the site during the major depositional phases (SI Figure 2.4). The lowermost samples are characterized by smaller pollen counts and low concentrations which steadily rise up to about 250 cm, where the samples yielded high pollen counts and a greater concentration of pollen. For much of the middle section of the deposits, the pollen are often damaged and although identified types are high, these tend to be types that are easily distinguished (Poaceae/Amaranthaceae). There’s a modest improvement in pollen preservation/concentration between 60-90 cm as well. What we do see in these samples are examples of types introduced by trade-wind activity (*Olea*-type & Pinaceae), riparian/estuarine trees/shrubs (*Avicennia, Syzygium*), and regional Sudanian woodland pollen types (*Acacia, Celtis, Trema*). By identifying components of the local vegetation and broader atmospheric processes, the palynological results provide some important context for the phytolith results.

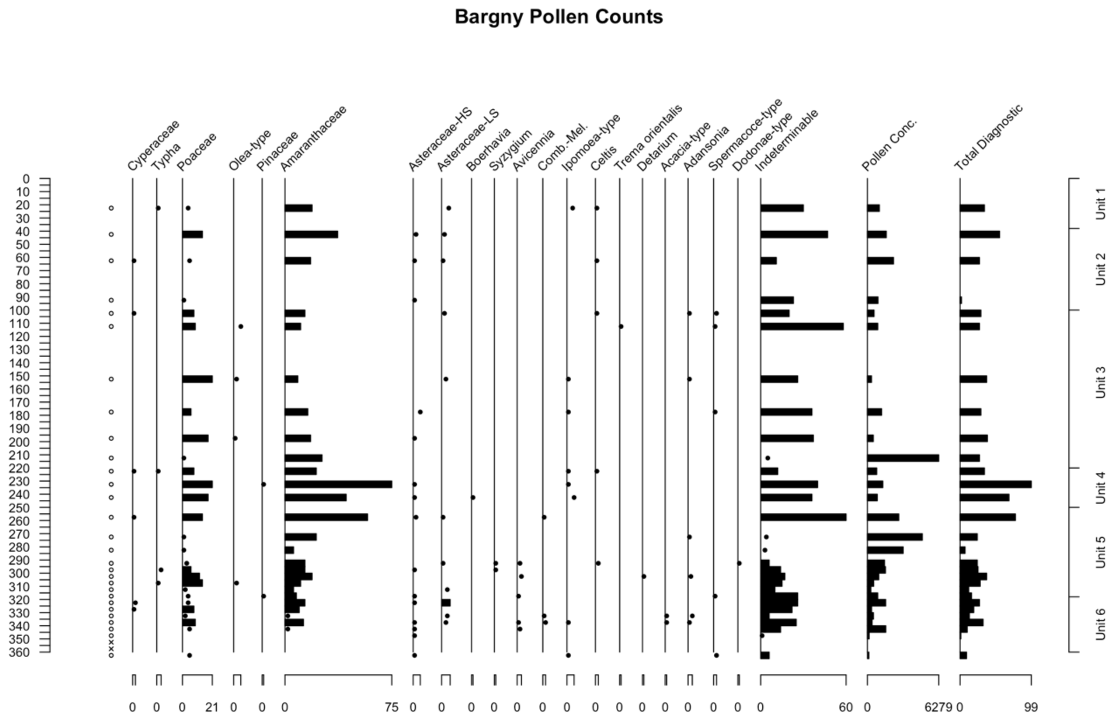

SI Figure 2.12: Pollen results as number of identifications per sample.

7. Summary Plots by Taxonomic Affiliation/Vegetation Cover

Assigning the phytolith results to taxonomic groups provides some limited insights (SI Figure 2.13). Grass phytoliths are dominant in the Bargny1 phytolith record and follow the overall trends seen in grass pollen. Panicoid grasses are somewhat more common in the lowermost sedimentary units and become rarer across the upper three units, while Chloridoid grasses are generally more common with peaks in Unit 4 and one sample in Unit 2. Although the sphereoid-echinate category might be artificially inflated by similar-looking sponge fossils, it could also represent more palm cover in Units 5 and 6. The representation of different pollen types primarily reflects preservation, but it shows two peaks in grass influx in Unit 5 and Unit 3 followed by peaks in xeric pollen types (predominantly Amaranthaceae) in Unit 4 and Unit 2. Trade wind activity is evident in Unit 6 and Unit 3, while wetland herbs and riparian shrubs/trees are most common in Units 5 and 6. Wetland herbs reappear at low frequencies in Unit 4, Unit 2, and Unit 1. Trees from the broader Sudaneo-Guinean zone are present in most of the samples.

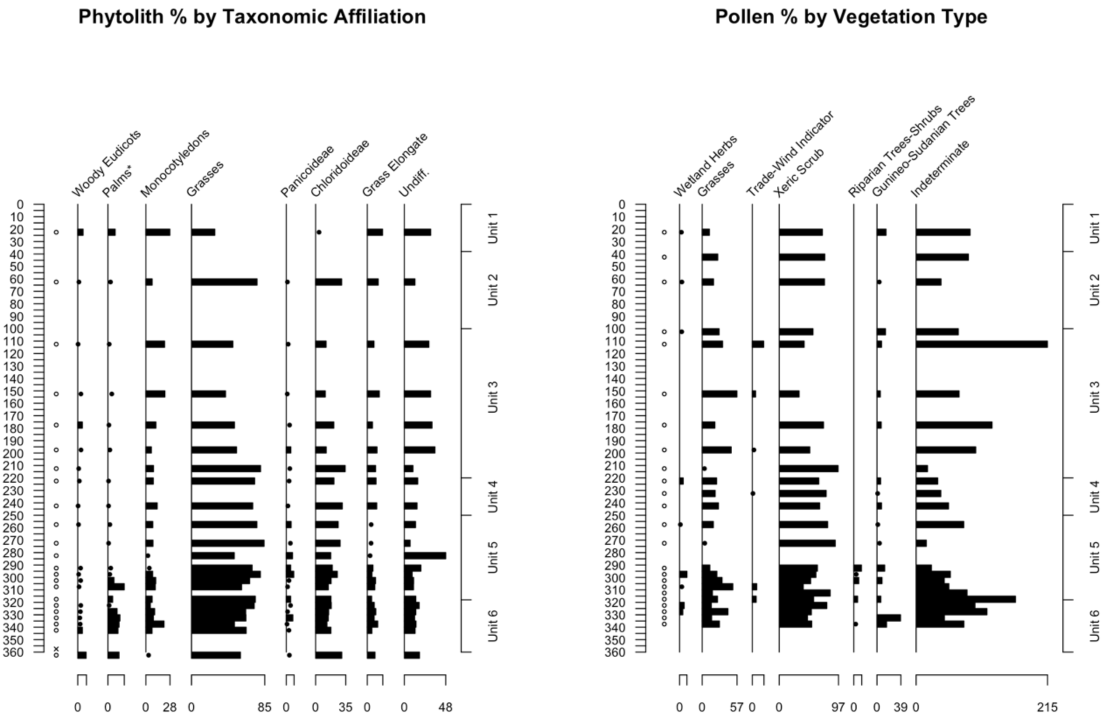

SI Figure 2.13: Phytolith and pollen percent by taxonomic affiliation and vegetation type, respectively.

8. Quantitative Comparisons with Surface Samples

Studies of surface soil phytoliths conducted by Bremond and colleagues[16,17] provide an important resource for evaluating archaeological phytolith assemblages. We use Principal Component Analysis (SI Figure 2.14) and Minimum Square-Chord Distances (SI Figure 2.15) to compare the phytolith spectra and to gain insights into the range of environments represented at Bargny 1. PCA was chosen because of the "predict" functionality in R, which is not available for other types of factor analysis (constrained correspondence, etc.). To look for changes in rainfall or the boundaries of major vegetation zones, we applied MSCD to establish the most similar sets of modern samples and plot their relevant climatic (rainfall) or geographic (latitude) representation across the depositional sequence.

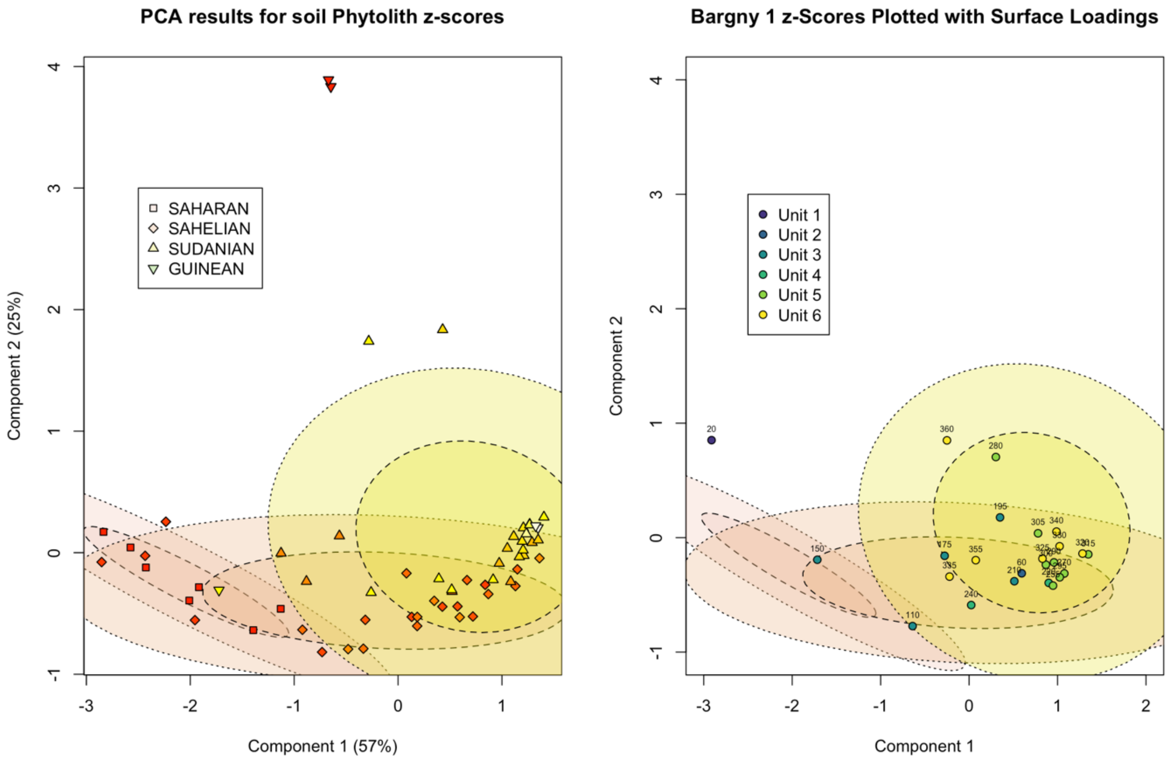

SI Figure 2.14: PCA of modern soil sample[16,17] (left) and Bargny 1 (right) results plotted using the same eigenvalues.

PCA shows the influence of rainfall on Bremond and colleagues[16,17] phytolith spectra. However, the individual vegetation zones are only generally resolved using these vectors. Ellipses (65% and 95%) are drawn around the important vegetation types near Bargny (Sahelian, Sudanian, and Saharan) to show the degree of overlap. The archaeological samples, when plotting using the eigenvectors from the original PCA, show a tight clustering with positive values on component 1 and low scores on component 2. Most of the samples fall within the 95% ellipse for Sahelian woodlands, but stratum 3 and 1 both yielded more negative component 1 loadings, consistent with drier conditions.

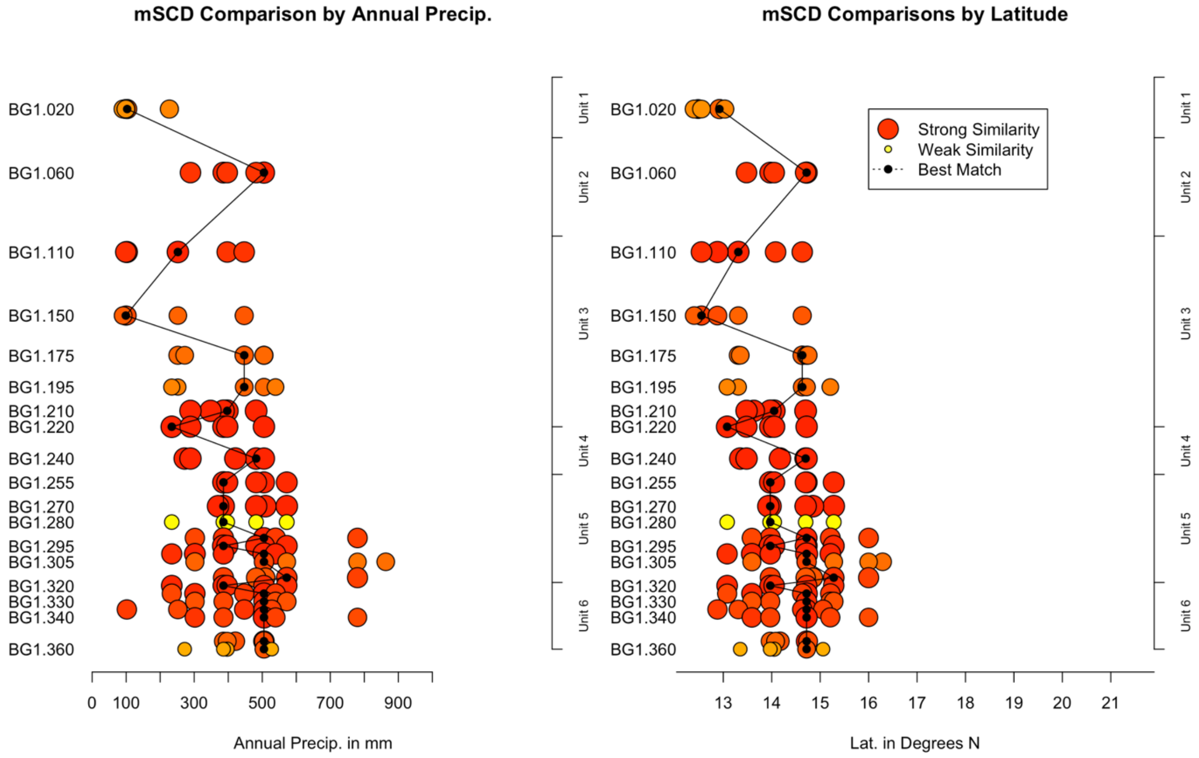

SI Figure 2.15 : Plots comparing Bargny samples with surface samples[16] using square-chord distances of scaled (z-score) diagnostic phytolith results. The five best matches are plotted by their current precipitation regime per sampled depth.

Comparing the Bargny 1 phytolith spectra with Bremond and colleagues[16,17] using MSCD produced some limited insights. Although the best-match approach shows some major downshifts in moisture (SI Figure 2.15) and reconstructed latitude in Unit 5 and Unit 4 (black dots with solid lines), the range of similar samples (yellow to red circles) is especially broad in the lowermost samples. In tandem with the presence of pollen from estuarine environments, which are not represented in Bremond and colleagues[16,17] surface sample studies, this signal may be interpreted two ways. First, riparian conditions may be bringing phytoliths from a larger source area to the site. Second, phytoliths primarily distinguish between two grass subfamilies in terrestrial settings, but the correlations between environmental conditions and the dominance of Chloridoid or Panicoid grasses does not hold up in riparian settings.

9. Phytolith Indices for Savanna Types and Grass Stress

We can also compare the values for indices used by Bremond and colleagues[16,17] to evaluate aridity/savanna type (Iph) and grass water stress (Fs) (SI Figure 2.8). These values do a good job of discriminating the boundaries between the Sudanian and Guinean zones (Iph values) and the Sahelian and Saharan zones (Fs). These values allow us to look for potential turnover between major vegetation formations.

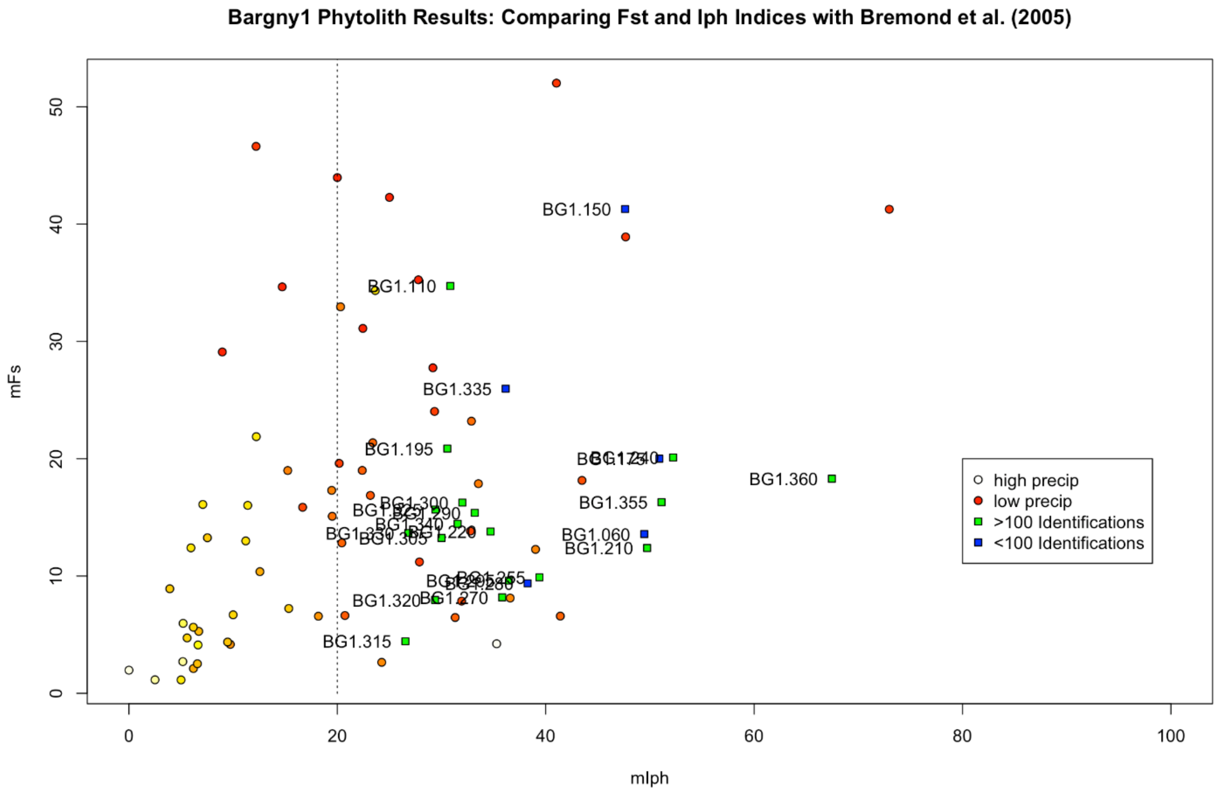

SI Figure 2.16: Comparison of long vs. short grass savanna index (Iph, x-axis) and Fan-shaped index (Fs, y-axis) results for Bargny samples (green and blue squares) with Bremond and colleagues[16] results from West African surface samples.

As with the MSCD results, the potential influence of estuarine conditions at the site might impact the phytolith signals in the lowermost units. This might explain why there is no complete turnover in Iph values, which never cross Bremond and colleagues[15] threshold between short and tall-grass savanna (SI Figure 2.16). The index for water stress (Fst) does show two prominent dry excursions at 110 and 150 cm, matching the mSCD, PCA, and palynological signals of aridity across Unit 3.

10. Synthetic Results

To provide an overview of the scale and direction of past vegetation change represented in the Bargny 1 deposits, we plotted the results for four different indices used above, grouping their results by stratum (SI Figure 2.17.). The indices include Fs, Iph, Grass-Amaranthaceae pollen, and wetland/riparian/mangrove pollen. As expected, the Grass/Amaranthaceae ratio is not especially informative, but the other indices show a clear turnover after Unit 4, when the depositional environment shifts from near-coastal estuarine conditions to halophytic dry coastal plain.

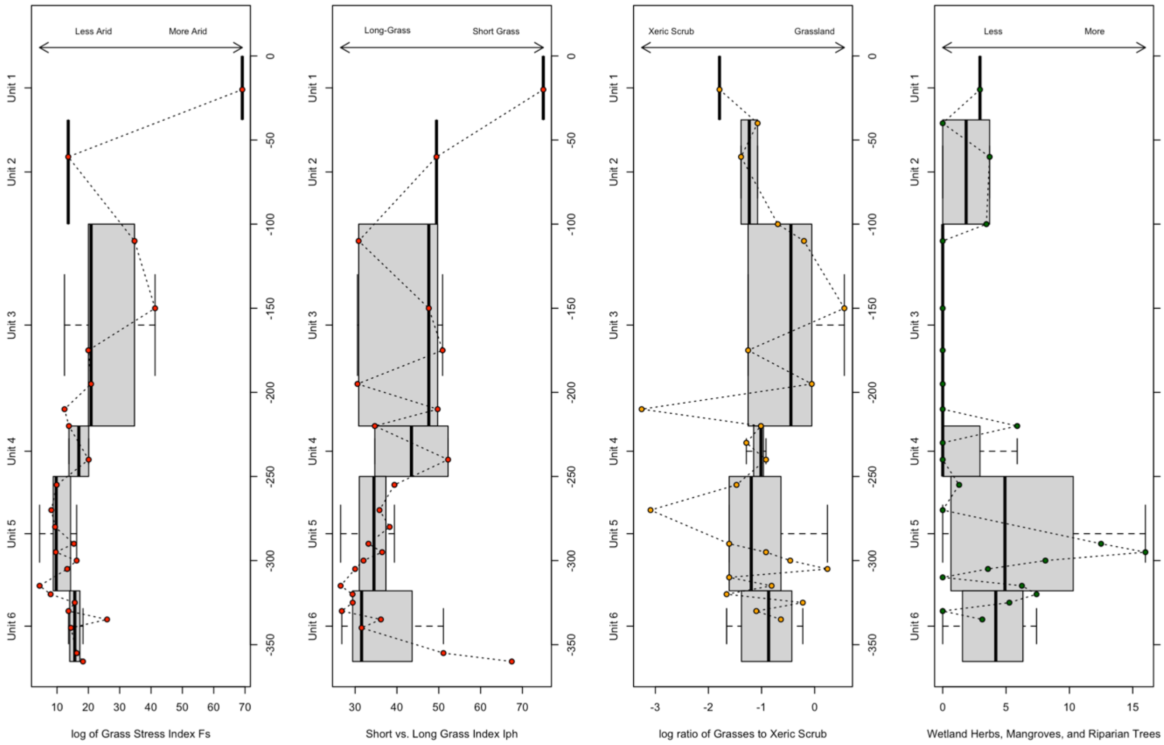

SI Figure 2.17: Box-and-whisker plots of phytolith indices and grouped pollen results by stratum alongside individual sample results (points). The mean value as the center line, upper (Q1) and lower (Q3) limits as the box, and minimum/maximum values as the whiskers.

We further synthesize the results (SI Figure 2.18) by plotting indices for water stress, short vs. long grass savanna, and riparian/estuarine pollen influx by stratum showing both the distribution of sample results (points) and their collective behavior (kernel density plots). In Unit 5 and Unit 6, riparian/estuarine conditions are associated with a distinct compression of variability in Iph and Fst values towards tall grass and low water stress conditions. By Unit 3 there is a turnover in the depositional environment and the phytolith signal shows more influence from short-grass savanna and higher water stress. This does not necessarily imply that climatic conditions are drier in Unit 3 compared with Unit 6. Given the chronology of the deposits, the riparian-influenced zone falls sometime during MIS-6, when regional conditions are arid[18,19] and jet-stream activity is high. The presence of Pinaceae pollen (an indicator of jet stream activity) in Bargny's MIS-6 deposits is consistent with this regional picture. Thus, it is possible that local hydrogeological factors are obscuring other signals of aridity during MIS-6 at Bargny. Sometime before the Last Glacial Maximum there is a turnover in the major depositional system at Bargny, although some local spring activity is still visible in the persistent presence of Typha.

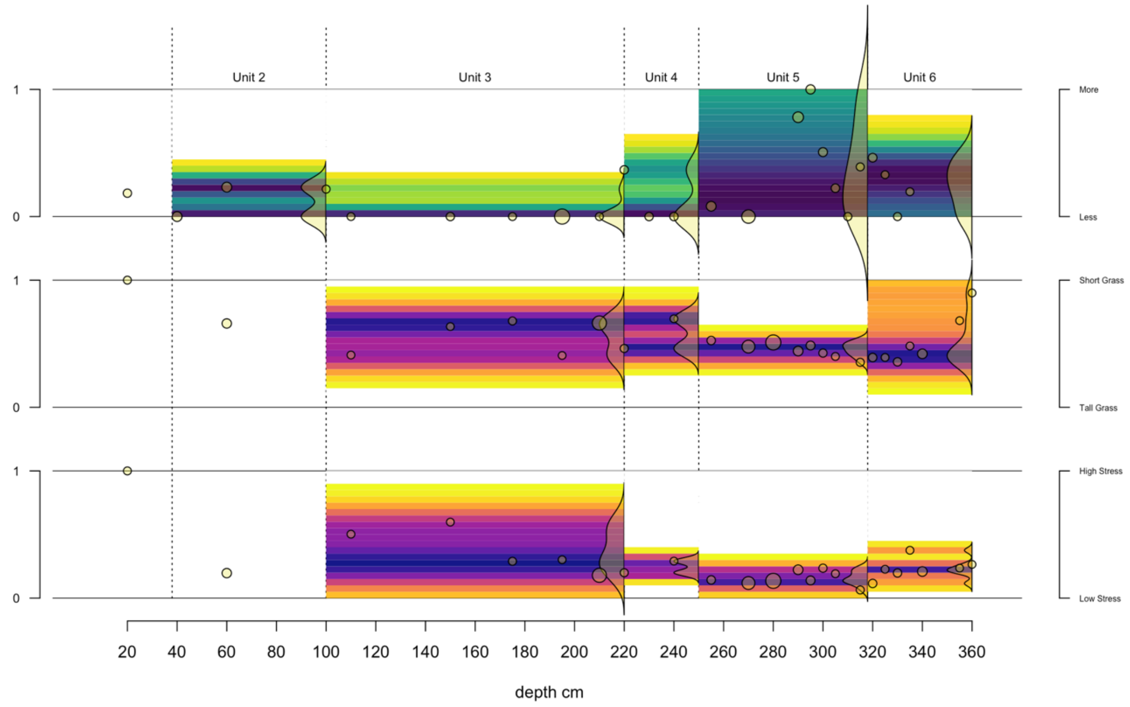

SI Figure 2.18: Synthetic results showing wetland, water stress, and precipitation proxies with kernel density plots for each stratum.

11. Regional Comparison and Context

Bargny currently lies at the margin of the Sahelian and Sudanian vegetation zones. Sahelian vegetation extends from 15º N - 18º N along the Atlantic coastline and is characterized by wooded grasslands and deciduous bushland, dominated by short-grasses from the Chloridoideae subfamily. Phytolith composition from surface samples shows greater contributions of flabellate and saddle-shaped morphotypes, reflecting the greater density of short-grasses and higher levels of water stress[16,17]. Sudanian woodlands occupy a narrow band of latitudes along the coastline near 15º N, but the latitudinal distribution progressively increases to below 10º N further inland. Annual grasses from the Panicoideae are dominant in the tall grass savanna communities in this region. Flabellate and saddle-shaped phytoliths are less common in surface samples from these environments[16].

The woody component of the Sahelian zone includes *Acacia* and *Mitracarpus*, both of which are common in the pollen spectra of surface samples from the region[19]. Woody vegetation makes a greater contribution to Sudanian vegetation cover. Typical woody trees include *Adansonia* and trees in the Combretaceae (e.g. *Terminalia*), but many species are shared with the Guinean rain forest zone which extends south along the Atlantic coast from 14º N. Common palynological signals of Sudanian-Guinean woody types are *Celtis*, *Elaeis guineensis*, *Alchornea*, and *Dodonaea*[19,20]. The grassy components of the Sudanian woodland and Guinean forest zones is also similar, generally showing low frequencies of flabellate and saddle-shaped phytoliths[16].

Studies of pollen[19] and phytoliths[16,17] from surface samples across west Africa give us some clear expectations for microfossil signals of past vegetation change at Bargny. Hooghiemstra and colleagues[19] identify the ratio of Amaranthaceae-Chenopodiaceae pollen and Poaceae pollen as a

signal of the Sahara's latitudinal position. Pollen transported from the montane and steppe regions of North Africa such as *Pinus*, *Artemisia*, and *Olea* are markers of trade wind intensity and the position of the Inter-Tropical Convergence Zone. Bremond and colleagues[15] used phytoliths from the same set of surface samples to identify a threshold of saddle-shaped phytolith frequencies for short- vs long-grass savanna cover (2.0 +/- 0.4), as well as documenting a relationship between the frequency of flabellate-shaped phytoliths and water stress.

The exception to the latitudinal zonation of vegetation types are estuarine, coastal, and riparian vegetation formations. Coastal vegetation types include mangroves dominated by *Rhizophora* and *Avicennia*, with the latter occurring as far north as 20ºN in relict communities[21]. Open, brackish wetlands located further inland where inundation is less frequent are dominated by *Avicennia*, *Typha*, and halophytic (salt-tolerant) grasses, while gallery forests along waterways show more Guinean components (*Alchornea*, *Macaranga*, *Uapaca*, *and Syzygium*).

Given the chronology of the Bargny 1 sedimentary sequence, we have high confidence that these results represent environmental conditions for portions of MIS-6 (Units 5-6), MIS-3 (Unit 3), and MIS-1 (Unit 1). Unit 4 (4) is not directly dated and may represent either MIS-4 or MIS-5. The Last Glacial Maximum is likely represented in the upper section of Unit 3 (III). Samples appear to be deposited predominantly during rebounds from colder extremes (MIS-6b, MIS-3).

Marine paleoecological[22–24] and paleoclimatic records[18] show dry conditions and a profound southward shift tropical African vegetation zones during MIS-6. Sea surface levels are low at this time, placing Bargny 1 at its greatest distance from the coast at this point. Comparisons with annual precipitation and vegetation types from surface samples[16,17] as well as Iph% and Fst values point in the opposite direction, showing similarities with less arid Sudanian vegetation types, low water stress, and a greater contribution of tall-grass (Panicoideae) savanna to the phytolith spectrum. This might be explained by the presence of Avicennia, Cyperaceae, Syzygium, and Typha in the pollen spectrum, all of which point to riparian/estuarine conditions. This may render the Iph% metric less meaningful in these samples, since halophytic grasses in coastal West Africa include both Panicoideae (Imperata, see [25]) and Chloridoideae (Sporobulis robustis, see [26]). Thus, it is possible that the relatively stable and less arid signal that persists across units 5 and 6 is linked with local hydrological conditions supporting halophytic and riparian/mangrove vegetation.

MIS-5 is a complex phase and is often subdivided into subphases. MIS-5 opens with warmer SSTs in the North Atlantic and weak trade winds between 130-115 ka, contributing to a rapid northward shift in vegetation boundaries[23,24] and maximum contributions of C3 plants in marine records[22]. From 115-74 ka, MIS-5 shows a general decrease in SSTs, but intermediate contributions of C4 plants to marine cores[22] and pollen records suggest intermediate positions of major vegetation zones[23].

There are no accepted direct dates linking the Bargny 1 deposits with MIS-5 or MIS-4. Unit 4 caps the archaeological deposits and its phytolith and pollen signals both point to drier conditions. Although the water stress index (Fst) is low, there is turnover to drier grasses dominated by Chloridoideae and identifiable pollen shows a stronger terrestrial signal dominated by Amaranthaceae-Chenopodiaceae. Low North Atlantic SSTs and intense trade wind circulation[18] contribute to a pronounced dry trend through MIS-4 (74-60 kya). This phase is missing from some of the benchmark marine pollen records from West Africa[23], but other records show maximum extension of C4 carbon sources[22] and low influx of pollen representing humid vegetation types[24].

The Last Glacial Maximum (MIS-2) is characterized by cooler NA SSTs and reduced extension of the ITCZ[27] from 24-11.6 kya. Dupont and Agwu[23] find a major extension of "grass-rich dry open forest" (ibid:169) at this time and Hooghiemstra and colleagues[19] find evidence for expansion of the Sahara at this time. Unit 3 broadly follows the trajectory of MIS-3 to MIS-2, moving from intermediate to maximum aridity by the upper samples, which may overlap with the Last Glacial Maximum. The turnover to warm NA SSTs and stronger monsoonal precipitation after the Younger Dryas (ca. 11.6 kya) is widely recognized as a major humid event in terrestrial records and marine records alike[20,27]. However, Dupont and Agwu[23] and Castaeñeda and colleagues[22] both find a more limited expansion of forest pollen and sources of C3 carbon in marine records at this time compared with prior warm/humid events in MIS-3 or MIS-5. Holocene conditions at Bargny are captured by two samples, showing a return to less arid conditions during the early Holocene (Unit 2) and rather dry conditions in the near-surface sediments.

Supplementary Information 3: Archaeological Comparisons and Refugia Analysis

Data used in comparisons between Bargny and other MSA assemblages across Africa dated to MIS 6 are shown in Table SI3.1, with data for comparisons between Barny and other dated MSA assemblages from West Africa shown in Table SI3.2

Blinkhorn and colleagues[28] have recently presented a model for the distribution of refugia for human populations in Africa during the Late Pleistocene. This has highlighted the presence of a region of West Africa in which modelled Late Pleistocene precipitation[29] has remained consistently within the 68% confidence interval of mean annual precipitation (248-1403 mm) associated with mobile hunter-gatherer population distributions[30]. Here, we repeat this analysis, focusing on the timeframe in which Middle Stone Age assemblages are presently known to occur in West Africa (160-11ka), and concentrating on the 68% confidence interval proposed by Blinkhorn and colleagues[28], which effectively encapsulated Late Pleistocene occupations of eastern Africa, as well as more broadly reflecting longer term MSA distribution patterns in the region during the Middle and Late Pleistocene, as identified by Timbrell and colleagues[31].

Overall, this analysis spanning 160-11ka chronologically extends the trends observed for the Late Pleistocene, highlighting a discrete region in West Africa that is persistently within the habitable precipitation bracket throughout this extended timeframe, termed here the Senegambian refugia (SI Figure 3.1). Whilst the overall size of the Senegambian refugia is comparable to that previously identified for the Late Pleistocene, E-W connectivity with eastern Africa is notably more limited. A N-S cline in precipitation is observed in the Senegambian refugia, with increased precipitation observed in the south, whereas E-W clines are observed for both standard deviation and coefficient of variation of precipitation is seen, suggesting greatest stability in precipitation occurs in the western part of the refugia. Tropical Xerophytic woodland is the most commonly occurring habitat type, with greater stability of open habitat types present in the northern half of the Senegambian refugia contrasting with higher amplitude of habitat change and diversity seen in the southern half.

Table SI3.1: Composition of Middle Stone Age assemblages from across Africa dating within Marine Isotope Stage 6

	Mid Age	N	E	Levallois Flake Technology	Levallois Blade Technology	Levallois Point Technology	Centripetal Technology	Blade Technology	Bipolar Technology	Platform Core Technology	Large Cutting Tools	Backing/ Microliths	Borers	Burins	Denticulates	Notches	Points	RT Bifacial	Scrapers
Benzu_3[32]	173	35.9	-5.4	0	1	0	1	0	0	1	0	0	0	0	0	1	1	0	1
Ifri n'Ammar_Lower OI (54-63) [33]	171	34.8	-3.1	0	1	0	1	1	0	0	0	0	0	0	1	1	1	0	1
Benzu_4[32]	170	35.9	-5.4	0	1	0	1	0	0	1	0	0	0	0	0	1	1	0	1
Benzu_5[32]	168	35.9	-5.4	0	1	0	1	0	0	1	0	0	0	0	0	1	1	0	1
PinaclePoint_PP13B_LC-MSA6[34,35]	163.5	-34.2	22.1	0	1	0	0	1	0	0	0	0	0	0	0	0	1	0	0
BorderCave_2015-2017/5BS[36]	161	-27.0	32.0	1	1	0	0	1	0	0	0	0	0	0	0	0	0	0	0
ElandsBayCave_2011/LowerMSA(Liam to Kirsten)	159.5	-32.3	18.3	0	1	0	1	1	1	0	0	0	0	1	1	0	1	0	1
PinaclePoint_PP13B_West_6[34,35]	159	-34.2	22.1	0	1	0	1	1	0	0	0	0	0	0	0	0	1	0	0
Florisbad_M[37]	157	-28.8	26.1	0	0	0	0	1	0	1	0	0	0	0	0	0	1	0	0
BouriA19BEX[38]	156.5	10.3	40.6	1	1	1	0	1	0	0	0	0	0	0	0	0	1	0	0
BouriA19HEX[38]	156.5	10.3	40.6	0	0	0	1	0	0	0	0	0	0	0	0	0	0	0	0
BouriA19Surf[38]	156.5	10.3	40.6	1	1	1	1	1	0	0	1	0	0	0	0	0	1	0	0
BouriA26ASurf[38]	156.5	10.3	40.6	1	1	1	1	1	0	0	0	0	0	0	0	0	1	0	0
BouriA26BSurf[38]	156.5	10.3	40.6	1	1	1	1	1	0	0	1	0	0	0	0	0	1	0	0
BouriA26CSurf[38]	156.5	10.3	40.6	0	0	0	1	1	0	0	0	0	0	0	0	0	0	0	0
BouriA29Surf[38]	156.5	10.3	40.6	1	1	1	1	1	0	0	1	0	0	0	0	0	1	0	0
Bargny 1	150	14.7	-17.2	1	1	1	1	1	0	1	0	0	0	0	0	0	1	0	1
Marmonet Drift_H2[39]	147	-0.8	36.2	0	0	0	0	0	0	0	0	0	1	1	0	0	0	0	1
Ifri n'Ammar_Upper OI (50-53) [33]	145	34.8	-3.1	0	1	1	1	1	0	1	0	0	0	0	1	1	1	0	1
Ifri n'Ammar_Occ Intermed. (43-49) [33]	138	34.8	-3.1	0	0	0	0	0	0	1	0	0	0	0	0	0	0	0	1
Wonderkrater_Area C[40]	138	-24.4	28.8	0	0	0	1	1	0	0	0	0	0	0	1	1	1	0	1
Haua Fteah_Deep Sounding (below XXXV) [41,42]	136	32.9	22.0	0	1	0	0	1	0	0	1	0	1	1	0	1	1	0	1
Rhafas_Layer 5[43,44]	135	34.6	-1.9	0	1	1	0	0	0	0	0	1	0	0	1	1	1	0	1
Ifri n'Ammar_Lower OS (35-42) [33]	130	34.8	-3.1	0	1	0	1	1	0	1	0	1	1	0	1	1	1	0	1

Table SI3.2: Composition of dated Middle Stone Age assemblages from West Africa. *Assemblages in which retouched tools are not reported.

	Mid Age	N	E	Levallois Flake Technology	Levallois Blade Technology	Levallois Point Technology	Centripetal Technology	Blade Technology	Bipolar Technology	Platform Core Technology	Large Cutting Tools	Backing/ Microliths	Borers	Burins	Denticulates	Notches	Points	RT Bifacial	Scrapers
Bargny 1	150	14.7	-17.2	1	1	1	1	1	0	1	0	0	0	0	0	0	0	0	1
Faleme Ravin Blanc 1[45]	126	13.9	-12.2	1	0	1	1	1	0	0	1	1	1	0	1	1	1	0	1
Ounjougou Kokolo 2[46]	62	14.5	-3.5	0	0	0	1	0	1	0	1	0	0	0	1	0	0	0	1
Tiémassas S7*[47,48]	62	14.1	-16.6	1	0	0	1	1	0	0	0	0	0	0	0	0	0	0	0
Ounjougou Kokolo 2[46]	59	14.5	-3.5	0	0	0	1	0	1	0	1	0	0	0	1	0	0	0	1
Ounjougou Oumounaama atelier*[46]	59	14.5	-3.5	0	0	0	0	0	1	0	0	0	0	0	0	0	0	0	0
Ounjougou Ravin Kondo[46]	58	14.5	-3.5	1	0	0	0	0	0	0	0	0	0	0	0	0	1	0	0
Ounjougou Vipere Low.* [46]	56	14.5	-3.5	0	0	0	1	0	0	0	0	0	0	0	0	0	0	0	0
Ounjougou Vipere upp/Vipere 3*[46]	56	14.5	-3.5	0	0	0	1	0	0	0	0	0	0	0	0	0	0	0	0
Tiémassas S6*[47,48]	44	14.1	-16.6	1	0	0	1	1	0	0	0	0	0	0	0	0	0	0	0
Ounjougou Ravin des draperies*[46]	43	14.5	-3.5	0	0	0	1	0	0	0	0	0	0	0	0	0	0	0	0
Ounjougou Oumounaama Butte[46]	42	14.5	-3.5	0	0	0	0	0	0	0	0	0	0	0	0	0	1	0	0
Ounjougou Songona 1	42	14.5	-3.5	0	0	0	0	0	0	0	0	0	0	0	0	0	1	0	0
Ounjougou Oumounaama Px[46]	40	14.5	-3.5	0	0	0	0	0	0	0	0	0	0	0	0	0	1	0	0
Bilma* [49]	38	10.2	-0.8	1	0	0	0	0	0	0	0	0	0	0	0	0	0	0	0
Birimi [49]	37	10.2	-0.8	1	0	1	1	1	1	0	1	0	1	0	1	1	1	0	1
Faleme Missira III[35]	37	13.9	-12.2	0	0	0	1	0	1	0	0	0	0	0	0	0	0	1	1
Ounjougou Coupe Franck low[46]	37	14.5	-3.5	0	0	0	1	0	1	0	0	0	0	0	0	0	1	0	0
Ounjougou Orosobo*[46]	37	14.5	-3.5	1	0	0	1	0	0	0	0	0	0	0	0	0	0	0	0
Ounjougou Orosobo 2*[46]	37	14.5	-3.5	1	0	0	0	0	0	0	0	0	0	0	0	0	0	0	0
Ounjougou Dandoli 1/3*[46]	33	14.5	-3.5	0	0	0	0	0	1	0	0	0	0	0	0	0	0	0	0
Ounjougou Dandoli 2*[46]	33	14.5	-3.5	0	0	0	0	0	0	0	1	0	0	0	0	0	0	0	0
Faleme Toumboura III[35]	32	13.9	-12.2	0	0	0	0	1	0	0	0	0	0	0	0	0	1	0	1
Ounjougou Coupe Franck upp.* [46]	31	14.5	-3.5	0	0	0	1	0	0	0	0	0	0	0	0	0	0	0	0

Ounjougou Kokolo 3[46]	30	14.5	-3.5	0	0	0	0	0	0	0	0	0	0	0	0	0	1	0	0
Ounjougou Sinkarma 1*[46]	28	14.5	-3.5	0	0	0	0	0	0	0	1	0	0	0	0	0	0	0	0
Faleme Toumboura II[35]	26	13.9	-12.2	0	0	0	0	0	0	0	0	0	0	0	1	1	0	0	1
Tiémassas S5[47,48]	26	14.1	-16.6	1	0	0	1	1	0	0	0	0	0	0	0	0	0	1	0
Laminia* [50]	23	12.6	-12.1	1	0	0	0	0	0	1	0	0	0	0	0	0	0	0	0
Ndiayène Pendao [51]	12	16.3	-15.4	1	0	1	0	1	0	0	1	0	0	0	1	0	1	1	0
Saxonomunya[37]	11	12.9	-11.4	1	0	0	1	0	0	1	0	0	0	0	1	1	1	0	1

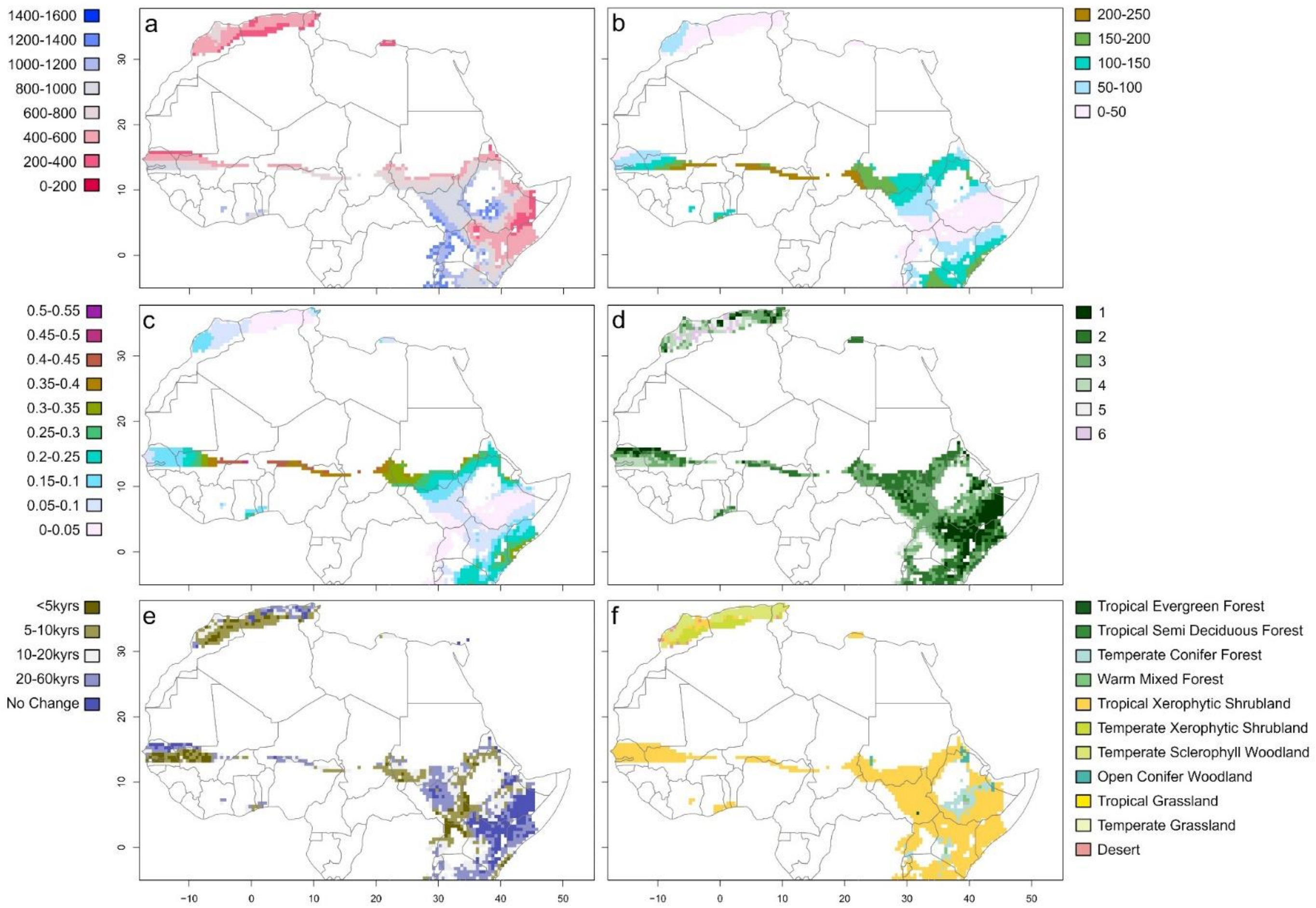

SI Figure 3.1: Maps of a) averaged mean annual precipitation (mm), b) standard deviation of mean annual precipitation (mm), c) coefficient of variation of mean annual precipitation, d) count of alternate biomes present, e) frequency of biome change, and f) modal biome constrained to regions in which mean annual precipitation remains within 68% confidence interval of mean annual precipitation associated with mobile hunter-gatherer population distributions (248-1403 mm) spanning the timeframe of MSA occupations of West Africa (160-11ka). These support the persistent habitability of the Senegambian refugia from the initial occupation at Bargny 1 to the onset of the Holocene and youngest MSA occupations at Saxomununya, and offer insight into the role of environmental stability on observed patterns of technological homogeneity.

Supplementary Information: References

1. Guerin, G., Mercier, N. & Adamiec, G. Dose-rate conversion factors: update. *Anc. TL* **29**, 5–8 (2011).
2. Aitken, M. *Introduction to optical dating: the dating of Quaternary sediments by the use of photon-stimulated luminescence*. (Clarendon Press, 1998).
3. Prescott, J. R. & Hutton, J. T. Cosmic ray contributions to dose rates for luminescence and ESR dating: Large depths and long-term time variations. *Radiat. Meas.* **23**, 497–500 (1994).
4. Bateman, M. D. & Catt, J. A. An absolute chronology for the raised beach and associated deposits at Sewerby, East Yorkshire, England. *J. Quat. Sci.* **11**, 389–395 (1996).
5. Murray, A. S. & Wintle, A. G. Luminescence dating of quartz using an improved single- aliquot regenerative-dose protocol. *Radiat. Meas.* **32**, (2000).
6. Murray, A. S. & Wintle, A. G. The single aliquot regenerative dose protocol : potential for improvements in reliability. *Radiat. Meas.* **37**, 377–381 (2003).
7. Bateman, M. . D. ., Frederick, C. . D. ., Jaiswal, M. . K. . & Singhvi, A. K. Investigations into the potential effects of pedoturbation on luminescence dating. *Quat. Sci. Rev.* **22**, 1169–1176 (2003).
8. Bateman, M. D. *et al.* Preserving the palaeoenvironmental record in Drylands: Bioturbation and its significance for luminescence-derived chronologies. *Sediment. Geol.* **195**, 5–19 (2007).
9. Bateman, M. D. *et al.* Detecting post-depositional sediment disturbance in sandy deposits using optical luminescence. *Quat. Geochronol.* **2**, 57–64 (2007).
10. Olley, J. M. *et al.* Optical dating of deep-sea sediments using single grains of quartz: a comparison with radiocarbon. *Sediment. Geol.* **169**, 175–189 (2004).
11. Murray, A. S. & Funder, S. Optically stimulated luminescence dating of a Danish Eemian coastal marine deposit: a test of accuracy. *Quat. Sci. Rev.* **22**, 1177–1183 (2003).
12. Galbraith, R. F. & Green, P. F. Estimating the Component Ages in a Finite Mixture. *Nucl. Tracks Radiat. Meas.* **17**, 197–206 (1990).
13. Lentfer, C. J. & Boyd, W. E. A comparison of three methods for the extraction of phytoliths from sediments. *J. Archaeol. Sci.* **25**, 1159–1183 (1998).
14. Lentfer, C. J. & Boyd, W. E. Simultaneous extraction of phytoliths, pollen and spores from sediments. *J. Archaeol. Sci.* **27**, 363–372 (2000).
15. Neumann, K. *et al.* International Code for Phytolith Nomenclature (ICPN) 2.0. *Ann. Bot.* **124**, 189–199 (2019).
16. Bremond, L., Alexandre, A., Hély, C. & Guiot, J. A phytolith index as a proxy of tree cover density in tropical areas: Calibration with Leaf Area Index along a forest-savanna transect in southeastern Cameroon. *Glob. Planet. Change* **45**, 277–293 (2005).
17. Bremond, L. *et al.* Phytolith indices as proxies of grass subfamilies on East African tropical mountains. *Glob. Planet. Change* **61**, 209–224 (2008).
18. deMenocal, P. B., Ruddiman, W. F. & Pokras, E. M. Influences of High- and Low-Latitude processes on African terrestrial climat: Pleistocene eolian records from Equitorial Atlantic Ocean Drilling Program Site 663. *Paleoceanography* **8**, 209–242 (1993).

19. Hooghiemstra, H., Lézine, A. M., Leroy, S. A. G., Dupont, L. & Marret, F. Late Quaternary palynology in marine sediments: A synthesis of the understanding of pollen distribution patterns in the NW African setting. *Quat. Int.* **148**, 29–44 (2006).

20. Lezine, A. -M, Turon, J. -L & Buchet, G. Pollen analyses off Senegal: Evolution of the coastal palaeoenvironment during the last deglaciation. *J. Quat. Sci.* **10**, 95–105 (1995).

21. Lézine, A. Evolution of the West African Mangrove During the Late Quaternary: A Review. *Géographie Phys. Quat. Evol.* **51**, 405–414 (1997).

22. Castañeda, I. S. *et al.* Wet phases in the Sahara/Sahel region and human migration patterns in North Africa. *Proc. Natl. Acad. Sci.* **106**, 20159–20163 (2009).

23. Dupont, L. M. & Agwu, C. O. C. Latitudinal shifts of forest and savanna in N. W. Africa during the Brunhes chron: further marine palynological results from site M 16415 (9°;N 19°W). *Veg. Hist. Archaeobot.* **1**, 163–175 (1992).

24. Lezine, A. M. & Casanova, J. Correlated oceanic and continental records demonstrate past climate and hydrology of North Africa (0-140 ka). *Geology* **19**, 307–310 (1991).

25. White, F. The vegetation of Africa: a descriptive memoir to accompany the UNESCO/AETFAT/UNSO vegetation map of Africa. (1983) doi:10.2307/2260340.

26. Hughes, R. H. *A directory of African weltands*. (IUCN, 1992).

27. DeMenocal, P., Ortiz, J., Guilderson, T. & Sarnthein, M. Coherent high- and low-latitude climate variability during the holocene warm period. *Science (80-.).* **288**, 2198–2202 (2000).

28. Blinkhorn, J., Timbrell, L., Grove, M. & Scerri, E. M. L. Evaluating refugia in recent human evolution in Africa. *Philos. Trans. R. Soc. B Biol. Sci.* **377**, (2022).

29. Krapp, M., Beyer, R. M., Edmundson, S. L., Valdes, P. J. & Manica, A. Terrestrial climate of the last 800,000 years. *Scientific Data* vol. 8 1–18 (2021).

30. Binford, L. *Constructing frames of reference: an analytical method for archaeological theory building using ethnographic and environmental data sets*. (University of California Press, 2001).

31. Timbrell, L., Grove, M., Manica, A., Rucina, S. & Blinkhorn, J. A spatiotemporally explicit paleoenvironmental framework for the Middle Stone Age of eastern Africa. *Sci. Rep.* **12**, 1–14 (2022).

32. Ramos, J. *et al.* The Benzú rockshelter: a Middle Palaeolithic site on the North African coast. *Quat. Sci. Rev.* **27**, 2210–2218 (2008).

33. ROCEEH Database (ROAD). Locality Ifri n Ammar. (2021).

34. Thompson, E., Williams, H. M. & Minichillo, T. Middle and late Pleistocene Middle Stone Age lithic technology from Pinnacle Point 13B (Mossel Bay , Western Cape Province , South Africa) q. *J. Hum. Evol.* **59**, 358–377 (2010).

35. Marean, C. W. *et al.* The stratigraphy of the Middle Stone Age sediments at Pinnacle Point Cave 13B (Mossel Bay, Western Cape Province, South Africa). *J. Hum. Evol.* **59**, 234–255 (2010).

36. Backwell, L. R. *et al.* New Excavations at Border Cave, KwaZulu-Natal, South Africa. *J. F. Archaeol.* **43**, 417–436 (2018).

37. Kuman, K. & Inbar, M. Palaeoenvironments and Cultural Sequence of the Florisbad Middle

Stone Age Hominid Site, South Africa. *J. Archaeol. Sci.* **26**, 1409–1425 (1999).

38. Clark, J. D. *et al.* Stratigraphic, chronological and behavioural contexts of Pleistocene Homo sapiens from Middle Awash, Ethiopia. *Nature* **423**, 747–752 (2003).

39. Slater, P. A. Change in lithic technological organization strategies during the Middle and Later Stone Ages in East Africa. **10301922**, 522 (2016).

40. Backwell, L. R. *et al.* Multiproxy record of late Quaternary climate change and Middle Stone Age human occupation at Wonderkrater, South Africa. *Quat. Sci. Rev.* **99**, 42–59 (2014).

41. Jacobs, Z. *et al.* The chronostratigraphy of the Haua Fteah cave (Cyrenaica, northeast Libya) — Optical dating of early human occupation during Marine Isotope Stages 4, 5 and 6. *J. Hum. Evol.* **105**, 69–88 (2017).

42. ROCEEH Database (ROAD). Locality Haua Fteah. (2021).

43. Doerschner, N. *et al.* A new chronology for rhafas, northeast Morocco, spanning the north African middle stone age through to the neolithic. *PLoS One* **11**, 1–34 (2016).

44. ROCEEH Database (ROAD). Locality Rhafas Cave. (2021).

45. Douze, K. *et al.* A West African Middle Stone Age site dated to the beginning of MIS 5 : Archaeology , chronology , and paleoenvironment of the Ravin Blanc I (eastern Senegal). *J. Hum. Evol.* **154**, 102952 (2021).

46. Chevrier, B. *et al.* Between continuity and discontinuity: An overview of the West African Paleolithic over the last 200,000 years. *Quat. Int.* **466**, 3–22 (2018).

47. Niang, K., Blinkhorn, J. & Ndiaye, M. The oldest Stone Age occupation of coastal West Africa and its implications for modern human dispersals: New insight from Tiémassas. *Quat. Sci. Rev.* **188**, 167–173 (2018).

48. Niang, K. *et al.* The Middle Stone Age occupations of Tiémassas, coastal West Africa, between 62 and 25 thousand years ago. *J. Archaeol. Sci. Reports* **34**, 102658 (2020).

49. Baluh, A. K. The Middle Stone Age in West Africa : Lithics from the Birimi Site in Northern Ghana. (University of South Carolina, 2017).

50. Scerri, E. M. L. *et al.* Continuity of the Middle Stone Age into the Holocene. *Sci. Rep.* **11**, 1–11 (2021).

51. Scerri, E. M. L., Blinkhorn, J., Niang, K., Bateman, M. D. & Groucutt, H. S. Persistence of Middle Stone Age technology to the Pleistocene/Holocene transition supports a complex hominin evolutionary scenario in West Africa. *J. Archaeol. Sci. Reports* **11**, (2017).
