# Supplementary material for: Longstanding behavioural stability in West Africa extends to the Middle Pleistocene at Bargny, coastal Senegal
Source: Nat Ecol Evol. 2023 May 4;7(7):1141–51. doi: 10.1038/s41559-023-02046-4 (PMC10333124; doi:10.1038/s41559-023-02046-4)
Supplement: Supplementary file 2 — Reporting Summary [file 41559_2023_2046_MOESM2_ESM.pdf]

nature portfolio

Corresponding author(s): J. Blinkhorn, K. Niang

Last updated by author(s): 08/02/2023

Reporting Summary

Nature Portfolio wishes to improve the reproducibility of the work that we publish. This form provides structure for consistency and transparency in reporting. For further information on Nature Portfolio policies, see our Editorial Policies and the Editorial Policy Checklist.

Statistics

For all statistical analyses, confirm that the following items are present in the figure legend, table legend, main text, or Methods section.

n/a	Confirmed	
☒	☐	The exact sample size (*n*) for each experimental group/condition, given as a discrete number and unit of measurement
☒	☐	A statement on whether measurements were taken from distinct samples or whether the same sample was measured repeatedly
☒	☐	The statistical test(s) used AND whether they are one- or two-sided<br>*Only common tests should be described solely by name; describe more complex techniques in the Methods section.*
☒	☐	A description of all covariates tested
☒	☐	A description of any assumptions or corrections, such as tests of normality and adjustment for multiple comparisons
☒	☐	A full description of the statistical parameters including central tendency (e.g. means) or other basic estimates (e.g. regression coefficient) AND variation (e.g. standard deviation) or associated estimates of uncertainty (e.g. confidence intervals)
☒	☐	For null hypothesis testing, the test statistic (e.g. *F*, *t*, *r*) with confidence intervals, effect sizes, degrees of freedom and *P* value noted<br>*Give P values as exact values whenever suitable.*
☒	☐	For Bayesian analysis, information on the choice of priors and Markov chain Monte Carlo settings
☒	☐	For hierarchical and complex designs, identification of the appropriate level for tests and full reporting of outcomes
☒	☐	Estimates of effect sizes (e.g. Cohen's *d*, Pearson's *r*), indicating how they were calculated

Our web collection on statistics for biologists contains articles on many of the points above.

Software and code

Policy information about availability of computer code

Data collection	*Provide a description of all commercial, open source and custom code used to collect the data in this study, specifying the version used OR state that no software was used.*
Data analysis	Data analysis was conducted using R; analytical code supplied as SI will be uploaded to Figshare as part of final submission

For manuscripts utilizing custom algorithms or software that are central to the research but not yet described in published literature, software must be made available to editors and reviewers. We strongly encourage code deposition in a community repository (e.g. GitHub). See the Nature Portfolio guidelines for submitting code & software for further information.

Data

Policy information about availability of data

All manuscripts must include a data availability statement. This statement should provide the following information, where applicable:

- Accession codes, unique identifiers, or web links for publicly available datasets
- A description of any restrictions on data availability
- For clinical datasets or third party data, please ensure that the statement adheres to our policy

The authors confirm that the data supporting the findings of this study are available within the article and its supplementary materials. Raw data and analytical code that supports the findings of this study that were supplied as SI will be uploaded to Figshare as part of final submission

Human research participants

Policy information about studies involving human research participants and Sex and Gender in Research.

Reporting on sex and gender	NA
Population characteristics	NA
Recruitment	NA
Ethics oversight	NA

Note that full information on the approval of the study protocol must also be provided in the manuscript.

Field-specific reporting

Please select the one below that is the best fit for your research. If you are not sure, read the appropriate sections before making your selection.

☐ Life sciences ☐ Behavioural & social sciences ☒ Ecological, evolutionary & environmental sciences

For a reference copy of the document with all sections, see nature.com/documents/nr-reporting-summary-flat.pdf

Ecological, evolutionary & environmental sciences study design

All studies must disclose on these points even when the disclosure is negative.

Study description	We present evidence for the late Middle Pleistocene MSA occupation of the West African littoral at Bargny, Senegal. OSL dating method replace the site at 150ka. The palaeoenvironmental data suggests relatively humid conditions and Mangrove ecotone. The description of the archaeological records is consistent with the early Middle stone Age technology as defined in large part of african continent. In contrast with other region, where different techno-typologial traditions emerge through the final Pleistocene, west African are shows an unexpected cultural stability until the Holocene.
Research sample	Samples of Pleistocene sediments were collected from the excavation site
Sampling strategy	Pleistocene sediments were collected at regular 0.05m intervals
Data collection	Pleistocene sediments were collected at regular 0.05m intervals
Timing and spatial scale	Fieldwork was undertaken at the excavation site in Dec 2017/Jan 2018
Data exclusions	No data were excluded
Reproducibility	The excavation site has subsequently been destroyed by quarrying prohibiting further investigation of the site
Randomization	NA
Blinding	NA

Did the study involve field work? ☒ Yes ☐ No

Field work, collection and transport

Field conditions	Bargny is located in the Sahel, the mean annual temperature is about 29° C, annual precipations vary beetween 400 and 600 mm
Location	Study took place in coastal Senegal in the locality of Bargny (14°41'N, 17°13W - altitude 13m)
Access & import/export	Fieldwork (including sampling) sampling were authorized by the Direction du Patrimoine culturel (DPC/NS 01-007 00237), 21st December 2017
Disturbance	The site is located in an old quarry. The preservation of the small section we excavated was due to the fact that until recently (1970's) the site location was used for mystic rituals. Our excavation interested a small surface (1 X 1.75m)

Reporting for specific materials, systems and methods

We require information from authors about some types of materials, experimental systems and methods used in many studies. Here, indicate whether each material, system or method listed is relevant to your study. If you are not sure if a list item applies to your research, read the appropriate section before selecting a response.

Materials & experimental systems

n/a	Involved in the study
☒	☐ Antibodies
☒	☐ Eukaryotic cell lines
☐	☒ Palaeontology and archaeology
☒	☐ Animals and other organisms
☒	☐ Clinical data
☒	☐ Dual use research of concern

Methods

n/a	Involved in the study
☒	☐ ChIP-seq
☒	☐ Flow cytometry
☒	☐ MRI-based neuroimaging

Palaeontology and Archaeology

Specimen provenance: Pollens and phytoliths were extracted from sediment samples recovered throuh Bargny section at each 5 cm interval. Fieldwork and sampling were authorized by the "Direction du patrimoine Culturel" (DPC/NS 01-007 00237).

Specimen deposition: *Indicate where the specimens have been deposited to permit free access by other researchers.*

Dating methods: Optically Stimulated Luminescence (OSL)

☐ Tick this box to confirm that the raw and calibrated dates are available in the paper or in Supplementary Information.

Ethics oversight: No ethical approval was requried

Note that full information on the approval of the study protocol must also be provided in the manuscript.
